# Forecasting stroke-like episodes and outcomes in mitochondrial disease

**DOI:** 10.1093/brain/awab353

**Published:** 2021-12-20

**Authors:** Yi Shiau Ng, Nichola Z Lax, Alasdair P Blain, Daniel Erskine, Mark R Baker, Tuomo Polvikoski, Rhys H Thomas, Christopher M Morris, Ming Lai, Roger G Whittaker, Alasdair Gebbels, Amy Winder, Julie Hall, Catherine Feeney, Maria Elena Farrugia, Claire Hirst, Mark Roberts, Charlotte Lawthom, Alexia Chrysostomou, Kevin Murphy, Tracey Baird, Paul Maddison, Callum Duncan, Joanna Poulton, Victoria Nesbitt, Michael G Hanna, Robert D S Pitceathly, Robert W Taylor, Emma L Blakely, Andrew M Schaefer, Doug M Turnbull, Robert McFarland, Gráinne S Gorman

**Affiliations:** 1 Wellcome Centre for Mitochondrial Research, Translational and Clinical Research Institute; NIHR Newcastle Biomedical Research Centre and Campus for Ageing and Vitality, Newcastle University, Newcastle upon Tyne NE2 4HH, UK; 2 Directorate of Neurosciences, Royal Victoria Infirmary, The Newcastle upon Tyne Hospitals NHS Foundation Trust, Newcastle upon Tyne NE1 4LP, UK; 3 Department of Neurosciences, NHS Highly Specialised Service for Rare Mitochondrial Disorders, Newcastle upon Tyne NE2 4HH, UK; 4 Campus for Ageing and Vitality, Newcastle Brain Tissue Resource, Newcastle University, Edwardson Building, Newcastle upon Tyne NE4 5PL, UK; 5 Translational and Clinical Research Institute, Newcastle University, Newcastle upon Tyne NE2 4HH, UK; 6 Institute of Neurological Sciences, Queen Elizabeth University Hospital, Glasgow G51 4TF, UK; 7 Trust Headquarters, One Talbot Gateway, Baglan Energy Park, Baglan, Port Talbot SA12 7BR, UK; 8 Greater Manchester Neuroscience Centre, Salford Royal NHS Foundation Trust, Manchester Academic Health Science Centre, Salford M6 8HD, UK; 9 Aneurin Bevan Epilepsy Specialist Team, Aneurin Bevan University Health Board, Newport, NP20 2UB, UK; 10 Department of Neurology, Sligo University Hospital, Sligo F91 H684, Ireland; 11 Department of Neurology, Queen’s Medical Centre, Nottingham NG7 2UH, UK; 12 Department of Neurology, Aberdeen Royal Infirmary, NHS Grampian, Aberdeen AB25 2ZN, UK; 13 Nuffield Department of Women’s and Reproductive Health, University of Oxford, Oxford OX3 9DU, UK; 14 Department of Paediatrics, Medical Sciences Division, Oxford University, Oxford OX3 9DU, UK; 15 Department of Paediatrics, The Children's Hospital, Oxford, OX3 9DU, UK; 16 Department of Neuromuscular Diseases, University College London Queen Square Institute of Neurology and The National Hospital for Neurology and Neurosurgery, London WC1N 3BG, UK

**Keywords:** prognostic modelling, neuropathology, MELAS, mitochondrial DNA (mtDNA), seizures

## Abstract

In this retrospective, multicentre, observational cohort study, we sought to determine the clinical, radiological, EEG, genetics and neuropathological characteristics of mitochondrial stroke-like episodes and to identify associated risk predictors.

Between January 1998 and June 2018, we identified 111 patients with genetically determined mitochondrial disease who developed stroke-like episodes. Post-mortem cases of mitochondrial disease (*n* = 26) were identified from Newcastle Brain Tissue Resource. The primary outcome was to interrogate the clinico-radiopathological correlates and prognostic indicators of stroke-like episode in patients with mitochondrial encephalomyopathy, lactic acidosis and stroke-like episodes syndrome (MELAS). The secondary objective was to develop a multivariable prediction model to forecast stroke-like episode risk.

The most common genetic cause of stroke-like episodes was the m.3243A>G variant in *MT-TL1* (*n* = 66), followed by recessive pathogenic *POLG* variants (*n* = 22), and 11 other rarer pathogenic mitochondrial DNA variants (*n* = 23). The age of first stroke-like episode was available for 105 patients [mean (SD) age: 31.8 (16.1)]; a total of 35 patients (32%) presented with their first stroke-like episode ≥40 years of age. The median interval (interquartile range) between first and second stroke-like episodes was 1.33 (2.86) years; 43% of patients developed recurrent stroke-like episodes within 12 months. Clinico-radiological, electrophysiological and neuropathological findings of stroke-like episodes were consistent with the hallmarks of medically refractory epilepsy. Patients with *POLG*-related stroke-like episodes demonstrated more fulminant disease trajectories than cases of m.3243A>G and other mitochondrial DNA pathogenic variants, in terms of the frequency of refractory status epilepticus, rapidity of progression and overall mortality. In multivariate analysis, baseline factors of body mass index, age-adjusted blood m.3243A>G heteroplasmy, sensorineural hearing loss and serum lactate were significantly associated with risk of stroke-like episodes in patients with the m.3243A>G variant. These factors informed the development of a prediction model to assess the risk of developing stroke-like episodes that demonstrated good overall discrimination (area under the curve = 0.87, 95% CI 0.82–0.93; *c*-statistic = 0.89). Significant radiological and pathological features of neurodegeneration were more evident in patients harbouring pathogenic mtDNA variants compared with *POLG*: brain atrophy on cranial MRI (90% versus 44%, *P* < 0.001) and reduced mean brain weight (SD) [1044 g (148) versus 1304 g (142), *P* = 0.005].

Our findings highlight the often idiosyncratic clinical, radiological and EEG characteristics of mitochondrial stroke-like episodes. Early recognition of seizures and aggressive instigation of treatment may help circumvent or slow neuronal loss and abate increasing disease burden. The risk-prediction model for the m.3243A>G variant can help inform more tailored genetic counselling and prognostication in routine clinical practice.

## Teaser

Ng *et al*. show that clinical, radiological and neuropathological characteristics of stroke-like episodes in mitochondrial disease are consistent with the hallmarks of medically refractory epilepsy. They develop a novel prediction model to identify the risk of stroke-like episodes in individuals with the m.3243A>G variant.

## Introduction

Stroke remains the second leading cause of death world-wide and the principal cause of serious long-term disability, with the prevalence of self-reported stroke in the general population estimated at 2.5%.^1^ Speed of accurate diagnosis and rapid delivery of appropriate therapies are central to modern stroke care models.^2^ Up to 30% of patients with suspected stroke have stroke ‘mimics’ representing a significant proportion of all acute hospital admissions.^3,4^ A discrete group of individuals with mitochondrial disease experience a stroke ‘mimic’, termed stroke-like episode, as part of mitochondrial encephalopathy, lactic acidosis, and stroke-like episodes (MELAS) syndrome.^5,6^ Stroke-like episodes among people with mitochondrial disease are often a devastating paroxysmal clinical event and a characteristic feature of MELAS syndrome.^7^

The first description of a fatal neurodegenerative syndrome including stroke-like episodes, ragged red fibres and lactic acidaemia was initially reported in 1975,^8^ with the acronym MELAS coined a decade later to encapsulate a distinct mitochondrial disease syndrome.^5^

The m.3243A>G pathogenic variant in the mitochondrial DNA (mtDNA)-encoded *MT-TL1* gene has been identified in 80% of MELAS syndrome patients.^9,10^ Identification of other pathogenic mtDNA variants^11-13^ and recessively inherited *POLG* variants^14–16^ have also emerged as rarer causes of MELAS. Predicting the risk of stroke-like episodes in mtDNA-related MELAS syndrome is further complicated by the peculiarities of mitochondrial genetics, including mtDNA heteroplasmy, threshold effect for biochemical and clinical manifestation, mtDNA copy number and mitochondrial genetic bottleneck.^17^ However, the extraordinary variability of phenotype and genotype in patients with mitochondrial disease, including those with MELAS syndrome, is recognized as a significant barrier to both the study and management of this condition, often delaying diagnosis and impacting negatively on patient care.^18,19^ Four decades since the first description of MELAS syndrome, the mechanistic basis^20,21^ and clinical diagnostic criteria of stroke-like episodes remain controversial.

Here we present the results of a multicentre, observational study evaluating the clinico-radiological, electrophysiological, laboratory, molecular and brain histopathological correlates to devise a new risk prediction model for stroke-like episodes in patients with genetically defined mitochondrial disease.

## Materials and methods

### Study population

Three centres (Newcastle upon Tyne, London and Oxford) constituting the UK National Health Service (NHS) Highly Specialised Service for Rare Mitochondrial Disorders of Adults and Children retrospectively collected clinical, demographic and neuropathological data on all cases of stroke-like episodes that occurred in children and adults (age range 1–72 years) with genetically confirmed mitochondrial disease between January 1998 and June 2018 inclusive. Data for patients without MELAS syndrome were retrieved from the UK Mitochondrial Disease Patient Cohort (REC reference number: 13/NE/0326, approved by the NRES Committee North East–Newcastle and North Tyneside 2) to permit survival analysis and construction of a risk prediction model.

### Study design

**Figure 1:**
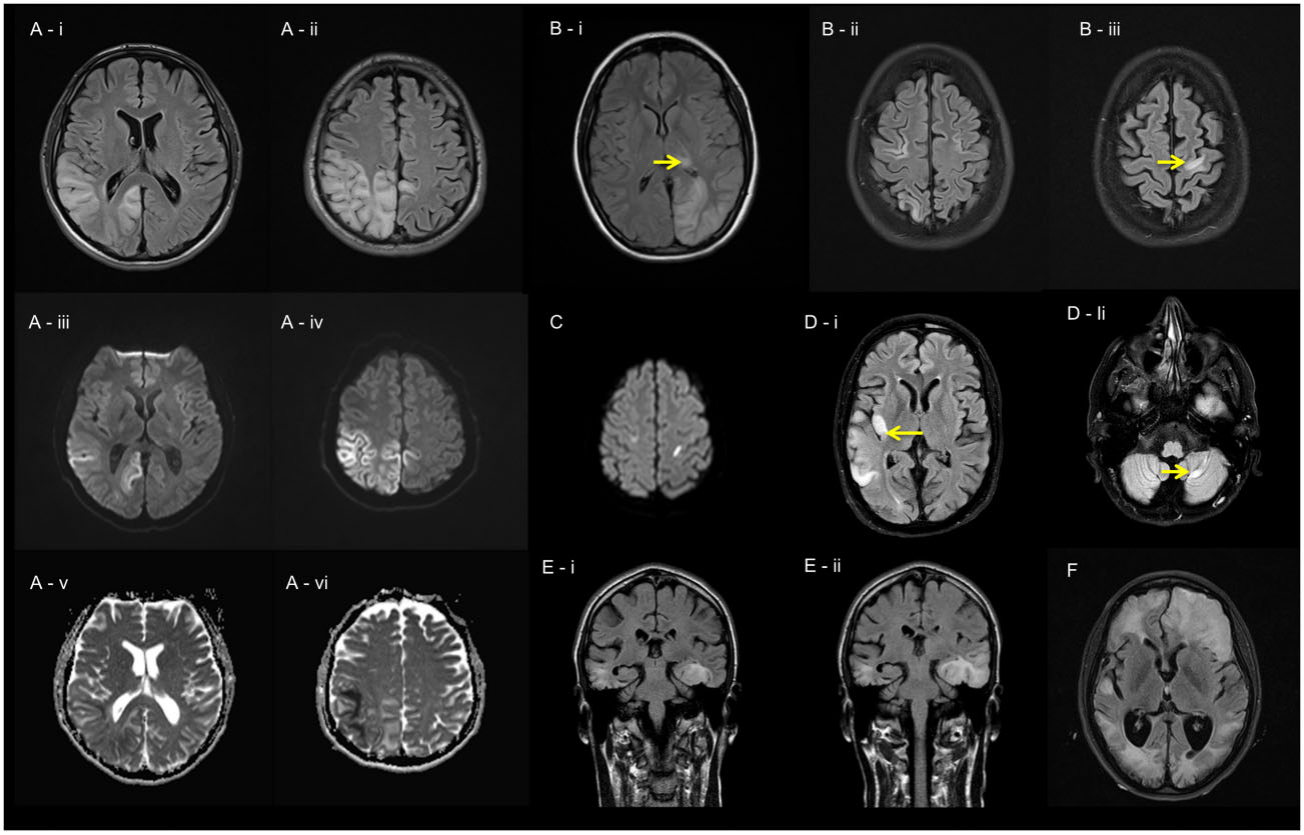
**Stroke-like lesions identified on MRI head.** Fluid-attenuated inversion recovery (FLAIR) sequence shows characteristic right occipital, temporal, parietal [**A**(**i**)] and left parietal [**A**(**ii**)] changes with restricted diffusion [**A**(**iii** and **iv**)] and mixed apparent diffusion coefficient (ADC) map changes [**A**(**v** and **vi**)] in a 20-year-old male with the m.3243A>G variant presenting with encephalopathy, occipital seizures, left hemianopia and apraxia. FLAIR sequence shows signal abnormalities involving left occipital lobe and pulvinar nucleus [**B**(**i**)] and multiple cortical signal abnormalities with restricted diffusion [**B**(**ii** and **iii**)] in a 25-year-old female with recessive *POLG* variants presenting with right hemianopia and status epilepticus. (**C**) Diffusion-weighted imaging identifies bilateral signal abnormalities approximate to precentral gyri in a 16-year-old female with recessive *POLG* variants presenting with refractory epilepsia partialis continua. FLAIR sequence demonstrates the phenomenon of cross-cerebellar diaschisis where signal abnormalities are present in right temporal and insular cortices [**D**(**i**)] and contralateral cerebellar hemisphere [**D**(**ii**)]. Coronal view of FLAIR sequence demonstrates bilateral temporal lobe changes [**E**(**i**)] at baseline, and the signal abnormalities in left mesial temporal lobe (hippocampus) evolved to involve the remaining temporal lobe on the interval scan a week later [**E**(**ii**)] in a 34-year-old male with the m.3243A>G variant presenting with headache, right hemianopia, global aphasia and focal motor seizures. Multiple, confluent signal abnormalities identified in both cerebral hemispheres (**F**) in a 24-year-old female with the m.3243A>G variant presenting with recurrent stroke-like episodes; only the signal abnormalities of left frontal lobe are associated with restricted diffusion (not shown) and there is evidence of generalized brain atrophy.

A stroke-like episode was defined as a discrete clinical event manifesting as focal neurological dysfunction, bilateral convulsion, altered consciousness, or any combination of these features.^7^ These paroxysmal events occurred in association with anatomically relevant acute (or subacute) cortical and subcortical^22^ neuroimaging abnormalities (Fig. 1 and examples of CT head changes are provided in Supplementary Fig. 1) and/or pathological EEG correlates.^7^ Participants with previous brain lesions or epileptogenic comorbidities considered unrelated to their primary mitochondrial disorder were excluded. The study was approved by the Newcastle and North Tyneside Local Research Ethics Committee (LREC 2005/202) and was conducted in accordance with the provisions of the Declaration of Helsinki and adhered to the Guidelines for Good Clinical Practice.

### Data collection

Clinical information including sex, age at disease onset and first stroke-like episode, duration between recurrent stroke-like episodes, survival status, clinical outcome and disease burden as assessed by the Newcastle Mitochondrial Disease Adult Scale (NMDAS)^23^ were collected. Clinical manifestations, laboratory data, neuroimaging findings, EEG studies and genetic test results were also collated.

### EEG data acquisition, analysis and interpretation

All EEG data were acquired by qualified EEG physiologists at the Department of Clinical Neurophysiology, Royal Victoria Infirmary, Newcastle upon Tyne Hospitals NHS Foundation Trust, UK. Reusable (autoclavable) gold cup electrodes were applied to the scalp in accordance with the international 10–20 system. A minimum of eight electrodes was applied in neonates, adults with challenging behaviours and in situations where large areas of scalp were unavailable for recording (e.g. in extensive craniotomy, by necessity, the number of electrodes had to be reduced). Approximately 20 min of EEG was acquired with the eyes closed throughout most of the recording but with brief epochs of eye opening and eye closure. Routine activation procedures included hyperventilation and photic stimulation.

EEG changes observed during episodes of encephalopathy, particularly metabolic encephalopathies, are generally non-specific in terms of aetiology and can range from an EEG with a normal alpha rhythm with intermixed theta and delta activity to generalized high-amplitude delta activity.^24,25^ The qualitative classification of EEG alterations was analysed based on methods described elsewhere.^26^ There are a number of recognized EEG signatures of seizure activity, but for the purposes of this study we limited our analysis to the presence or absence of lateralised periodic discharges as previously defined.^27^

### Neuropathological analysis

Neuropathological reports were reviewed from 26 patients (16 with documented antemortem stroke-like episodes). We compared brain weights of mitochondrial disease cases to those of every control case donated to Newcastle Brain Tissue Resource between January 2010 and July 2019. Formalin-fixed paraffin-embedded brain tissues from 10 patients with clinically and genetically defined mitochondrial disease and 10 cognitively normal, aged-matched controls were acquired from Newcastle Brain Tissue Resource (Supplementary Table 1). Tissue processing and general neurohistopathological staining was performed as previously described.^28,29^

#### Immunofluorescence and confocal microscopy for identification of OXPHOS subunits in microvessels and neurons

A quadruple immunofluorescence assay was developed to label oxidative phosphorylation (OXPHOS) subunits NDUFB8 and COX1 and mitochondrial mass marker porin in conjunction with α-smooth muscle actin (α-SMA) or GLUT-1 for smooth muscle (arterioles) and endothelial (capillaries) cells, respectively. Immunofluorescence was performed on 5-μm formalin-fixed paraffin-embedded sections of cerebellum, temporal and occipital cortices as previously described.^30^ The primary and secondary antibodies used are given in Supplementary Table 2.

Twenty cortical arterioles and capillaries per brain region were randomly selected on the basis of their immunoreactivity for α-SMA or GLUT-1, respectively, and imaged using a confocal microscope (Nikon A1R) as previously described.^31^ Imaging was performed at ×180 magnification (60× objective with 3× electronic zoom) and *z* stacking (0.175-µm increments). Laser settings were constant throughout imaging.

#### Quantification of OXPHOS subunit expression and deficiency

Volocity imaging software (v.6.1.1., Perkin Elmer) was used to identify arterioles and capillaries as ‘objects’ based on α-SMA or GLUT-1 immunoreactivity. Microvascular mitochondria were detected based on their porin immunoreactivity localised within either α-SMA or GLUT-1. The mean optical densities for porin, NDUFB8 and COX1 were then derived and used to determine *z-*scores as previously described.^31^ Percentage levels of OXPHOS deficiency could be determine from *z-*scores lower than −3.

### Statistical analyses

Statistical analyses were performed with the use of IBM SPSS Statistics software (version 23) and R programming language (version 3.5).^32^ Continuous data were presented as mean with SD; 95% CI were calculated where appropriate. Statistical significance was defined as *P* ≤ 0.05; where appropriate, *P*-values were adjusted for multiple comparisons using the Benjamini–Hochberg method.^33^ Pearson chi-squared test was performed to examine relationships between categorical variables including clinical features associated with stroke-like episodes, neuroimaging, EEG and neuropathological findings between mtDNA and *POLG* variants. Minimal prevalence of MELAS syndrome was calculated for North East England using data from UK Mitochondrial Disease Patient Cohort^6^ and population estimates from the 2011 UK Census.^34^

#### Developing a risk prediction model for the m.3243A>G variant

Survival curves were calculated and presented using the survival package within R.^35^ Putative predictors of stroke-like episodes were identified from the literature and clinical experience. Univariate analysis with Cox proportional hazards regression was then used to suggest significant predictors as candidates for a multivariate model. After confirming the assumptions of proportional hazards using Schoenfeld residuals, a multivariable Cox proportional hazards model usin a backward stepwise elimination approach (with change in Akaike Information Criterion for successive models as a threshold) was used to identify the variables that contribute to the final scoring system. Following the methodology described by Galovic and co-workers,^36^ we derived a three-tier risk model of developing stroke-like episodes for carriers of the m.3243A>G variant, and applied the receiver-operating characteristics curve techniques to evaluate the usefulness of our risk prediction model.

#### Andersen–Gill model

An Andersen–Gill model was used to analyse the time gaps between stroke-like-episodes.^37^ As we have no entry point for individuals, we took the first stroke-like-episode as a starting point and analysed the time to subsequent stroke-like-episodes. The model assumes that all events are ordered and equivalent.

### Data availability

The data that support the findings of this study are available from the corresponding author (G.S.G.) and first author (N.Z.L.), upon reasonable request.

## Results

### Genetic analysis

Between January 1998 and June 2018, a total of 111 patients with genetically confirmed mitochondrial disease and stroke-like episodes were identified. The most common causative pathogenic defect was the m.3243A>G (*MT-TL1*) variant (*n* = 72; 66%), followed by recessive *POLG* variants (*n* = 22; 20%) and 11 other rarer pathogenic mtDNA variants (*n* = 17; 14%). The other mtDNA variants were m.3764C>G (*MT-ND1*) (*n* = 1), m.5543T>C (*MT-TW*) (*n* = 1), m.7541T>C (*MT-TD*) (*n* = 1), m.8344A>G (*MT-TK*) (*n* = 1), m.10010T>C (*MT-TG*) (*n* = 1), m.10158T>C (*MT-ND3*) (*n* = 1), m.12147G>A (*MT-TH*) (*n* = 1), m.12770A>G (*MT-ND5*) (*n* = 1), m.13094T>C (*MT-ND5*) (*n* = 3), m.13513G>A (*MT-ND5*) (*n* = 4) and m.14430A>G (*MT-ND6*) (*n* = 1).

Twelve pathogenic *POLG* variants were identified in 23 individuals: p.Leu304Arg (*n* = 2), p.His360Asp (*n* = 1), p.Leu411Pro (*n* = 1), p.Ala467Thr (*n* = 27), p.Arg574Gln (*n* = 1), p.Pro587Leu/p.Pro589Thr (in *cis*) (*n* = 1), p.Arg597Trp (*n* = 1), p.Thr606Alafs*64 (*n* = 1), p.Arg627Gln (*n* = 1), p.Trp748Ser (*n* = 7), p.Gly848Ser (*n* = 1) and p.Thr914Pro. Homozygosity for the p.Ala467Thr variant was present in nine individuals (39%).

### Clinical characteristics of stroke-like episodes

Description on 229 stroke-like episodes were available [median (range), 2 (1–5)] with 63% of available data demonstrating radiological correlates (Table 1). There were no statistical differences in clinical, radiological and EEG data between cases of m.3243A>G and other mtDNA pathogenic variants (*P >* 0.05) and therefore they were analysed collectively as a single ‘mtDNA’ category. A total of 60 patients were female; mean (SD) age at last follow-up was 38.3 (16.2) years. There was a higher proportion of women in the *POLG* category compared to mtDNA [18/23 (78%) versus 42/88 (48%), *P* = 0.009]. The age of first stroke-like episode was available for 105 patients [mean (SD) age; range, 31.8 (16.1); 0.6–71]. Patient’s sex had no impact on age of first stroke-like episode or age at last follow-up. A total of 35 patients (32%) presented with their first stroke ≥40 years of age.

**Table 1:** Summary of the differences between mtDNA- and *POLG*-related stroke-like episodes

	mtDNA^a^	*POLG*	*P-*value
**Demographic data**			
*n*	88	23	–
Male:female	46:42	5:18	**0.009**
No. of family pedigrees	80	23	–
Mean age of first stroke-like episode (SD) [95% CI]	35.2 (14.6) [32.0–38.4]	18.3 (25.6) [11.2–25.4]	**<0.001**
Mean age of last follow-up (SD) [95% CI]	42.6 (13.8) [39.6–45.6]	22 (14.7) [15.5–28.5]	**<0.001**
Death	34 (39%)	17 (74%)	**0.017**
Mean age of death (SD) [95% CI]	46.5 (14.4) [41.2–51.8]	23 (17.5) [12.9–33.1]	**<0.001**
**Features of stroke-like episodes**			
Headache	51/68 (75%)	9/12 (75%)	0.894
Nausea/vomiting	15/29 (52%)	5/7 (71%)	0.500
Positive visual^b^	37/67 (55%)	7/17 (41%)	0.329
Negative visual	42/72 (58%)	10/19 (53%)	0.628
Focal weakness	30/65 (46%)	6/14 (43%)	0.704
Dysphasia	28/55 (51%)	1/11 (9%)	**0.048**
Sensory disturbance	14/46 (30%)	3/14 (21%)	0.689
Acute hearing loss	8/46 (17%)	0/10	0.286
Confusion/drowsiness	57/68 (84%)	12/18 (67%)	0.234
Neuropsychiatric^c^	29/53 (55%)	4/13 (31%)	0.263
Seizures			
Motor seizures	60/95 (63%)	27/28 (96%)	**0.007**
Occipital seizures	38/73 (52%)	7/21 (33%)	0.234
Status epilepticus	19/87 (22%)	27/28 (96%)	**0.007**
**Neuroimaging changes**			
Frontal	26/113 (23%)	13/32 (41%)	0.051
Insular	24/113 (21%)	1/32 (3%)	**0.042**
Parietal	80/113 (71%)	16/32 (50%)	0.053
Temporal	90/113 (80%)	5/32 (16%)	**0.004**
Occipital	86/113 (76%)	20/32 (63%)	0.167
Thalamus	7/113 (6%)	11/32 (34%)	**0.004**
Cross-cerebellar	13/113 (12%)	8/32 (25%)	0.139
**EEG findings (acute)**			
Encephalopathy	114/118 (97%)	65/89 (78%)	**0.036**
Epileptic discharge^d^	66/120 (55%)	67/89 (75%)	**0.002**
PLEDs	7/35 (20%)	21/56 (38%)	0.163

*P*-values were adjusted where appropriate for multiple comparisons. Values in bold represent those that reach significance, *P* > 0.05. ADC = apparent diffusion coefficient; BG = basal ganglia; PLED = periodic lateralizing epileptic discharge.

a The most common pathogenic mtDNA variant associated with stroke-like episodes is the m.3243A>G variant (72/88; 82%). There are no statistical differences in clinical, radiological and EEG data between cases of m.3243A>G and other mtDNA pathogenic variants, and therefore they are analysed collectively as one ‘mtDNA’ category.

b The details of positive visual symptoms are catalogued in Supplementary Table 3.

c The range of neuropsychiatric symptoms included agitation and aggressiveness, severe anxiety, psychosis and behavioural changes reported by the family.

d More details about the location of epileptic discharge are available in Supplementary Table 7.

The majority of mtDNA-related MELAS cases (69/75; 91%) had exhibited antecedent symptoms suggestive of a mitochondrial disorder, whereas 45% (9/20) of *POLG* patients were considered asymptomatic before presenting with their first stroke-like episode (*P* = 0.005). Sensorineural hearing loss [45/74 (61%) versus 0%, *P* = 0.005] preceding a stroke-like episode was more commonly associated with patients harbouring a pathogenic mtDNA variant while preceding ataxia was more frequently identified in patients with *POLG* variants [4/74 (30%) versus 6/20 (5%), *P* = 0.007] (Table 2).

**Table 2:** Clinical manifestations before the emergence of first stroke-like episode

	mtDNA^a^	*POLG*	*P-*value
No pre-existing symptom	6/75 (9%)	9/20 (45%)	**0.005**
Deafness	45/74 (61%)	0/20	**0.005**
Ataxia	4/74 (5%)	6/20 (30%)	**0.007**
Headache	16/73 (22%)	0/20	0.053
Diabetes mellitus	20/75 (27%)	1/20 (5%)	0.076
Renal	6/74 (8%)	0/20	0.269
Gut dysmotility	10/73 (14%)	1/20 (5%)	0.318
Seizures	10/74 (14%)	3/20 (15%)	0.864
Exercise intolerance/myopathy	6/73 (8%)	0/20	0.269
Cardiac	4/73 (6%)	0/20	0.318
Renal	6/74 (8%)	0/20	0.269

*P-*values were adjusted where appropriate for multiple comparisons.

a There are no statistical differences in clinical data between cases of m.3243A>G and other mtDNA pathogenic variants, and therefore they are analysed collectively as one ‘mtDNA’ category.

The most common symptoms associated with stroke-like episodes were confusion/drowsiness (69/86; 80%) followed by headache (60/80; 75%) and motor seizures (87/123; 71%) (Table 1 and Supplementary Fig. 2). Visual symptoms most often manifested as either positive (44/84; 57%) or negative (52/91; 57%) visual phenomena (ranging from elementary visual hallucinations to visual loss; Supplementary Table 3). Focal onset status epilepticus was identified in almost all stroke-like episodes in patients with *POLG* variants compared to about a fifth of mtDNA-related MELAS syndrome [27/28 (96%) versus 19/87 (22%), *P* = 0.007]. Dysphasia was significantly more common in mtDNA-related mitochondrial disease compared to *POLG*-related disease [28/55 (51%) versus 1/11 (9%), *P* = 0.048; Table 1]. The mean age of last follow-up was significantly younger and the overall disease burden measured by NMDAS was significantly higher in patients with stroke-like episodes compared to those without stroke-like episodes irrespective of the genetic defects (Supplementary Tables 4 and 5).

### Neuroimaging findings

Cranial MRI scans (*n* = 144) performed during stroke-like episodes were available for analysis. Peri-ictal abnormalities in the temporal lobe [90/113 (80%) versus 5/32 (16%), *P* = 0.004] and insular cortex [24/113 (21%) versus 1/32 (3%), *P* = 0.042] were significantly more common in the mtDNA group compared to *POLG* cases, whereas thalamic involvement was less commonly observed in the mtDNA group [7/113 (6%) versus 11/32 (34%), *P* = 0.004; Fig. 1 and Table 1]. None of the subcortical white matter changes occurred in isolation. Cross-cerebellar diaschisis was identified in 11% of all scans, with no statistical difference between mtDNA and *POLG* cases [13/113 (12%) versus 8/32 (25%), *P* = 0.139]. Peri-ictal MRI changes were identified on all MRI head scans of mtDNA mutation cases, while up to 16% of *POLG* scans did not identify any acute peri-ictal signal abnormality. MRI thalamic [12/46 (26%) versus 3/69 (4%), *P* = 0.004] and cross-cerebellar diaschisis [12/46 (26%) versus 6/69 (9%), *P *= 0.028] lesions were statistically more common in patients manifesting with status epilepticus compared to those not presenting in status epilepticus during a stroke-like episode (Supplementary Table 6). Imaging appearances of cerebral and cerebellar atrophy were significantly more evident in mtDNA compared to *POLG* cases [95/105 (90%) versus 12/27 (44%); *P <* 0.001 and 95/103 (92%) versus 16/28 (57%); *P <* 0.001, respectively; Supplementary Fig. 3].

### EEG findings

EEG data (*n* = 260) were available for analysis in 50 patients (m.3243A>G, *n* = 30; *POLG*, *n* = 13; others, *n* = 7). Eighty per cent of EEGs (*n* = 208) were performed during acute hospital admissions. Encephalopathic changes were more commonly identified in mtDNA than *POLG*-related MELAS syndrome [114/118 (97%) versus 65/89 (78%), *P* = 0.036]. Epileptic discharges were more frequently captured in *POLG* than mtDNA variants [67/89 (75%) versus 66/120 (55%), *P* = 0.002; Table 1] and more commonly identified in the posterior region in *POLG* compared to mtDNA cases [38/89 (43%) versus 23/120 (19%), *P <* 0.001; Supplementary Table 7]. Periodic lateralizing epileptic discharges were identified in 34% of all acute EEGs (28/91) irrespective of genotype and presence of clinical status epilepticus. Six episodes of epilepsia partialis continua were documented clinically without EEG correlates in *POLG* cases.

### Survival analysis

**Figure 2:**
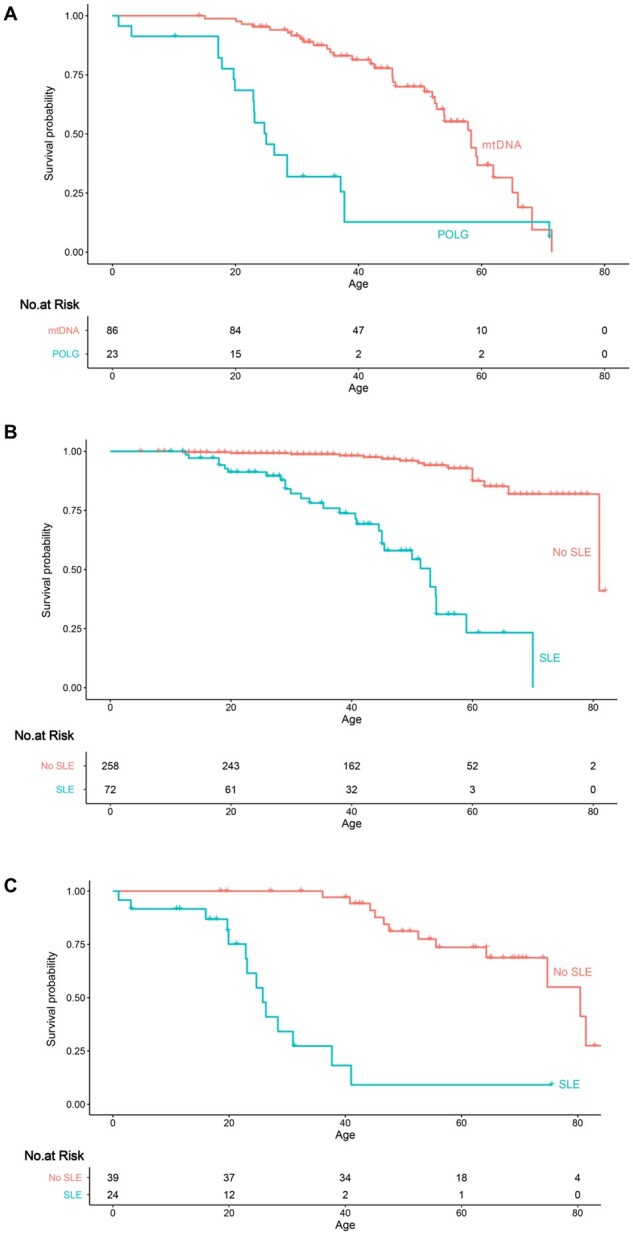
**Kaplan–Meier Estimates of Survival.** (**A**) The number of patients affected by stroke-like episodes at risk of death in both mtDNA and *POLG* groups. (**B**) The number of patients at risk of death in all individuals who harboured the m.3243A>G variant. (**C**) The number of patients at risk of death in all individuals who harboured the recessive *POLG* variants.

A minimum prevalence of 0.42 (95% CI 0.38–0.46) per 100 000 was calculated for patients experiencing stroke-like episodes in in the general population of North East England. *POLG* patients in this study had a significantly higher risk of early death [mean age (SD), 20.4 (10.6); hazard ration (HR) 3.9, 95% CI 2.2–7.0, *P <* 0.001] than individuals affected by m.3243A>G [mean age (SD), 45.8 (14.9] or other pathogenic mtDNA variants [mean age (SD), 48 (11.9)]. Moreover, stroke-like episodes were a significant predictor of early death in both m.3243A>G (HR 12.3, 95% CI 5.8–26.5, *P <* 0.001; Supplementary Table 8) and *POLG* (HR 11.6, 95% CI 4.7–28.2, *P <* 0.001) patients (Fig. 2).

### Risk prediction of stroke-like episodes associated with m.3243A>G

**Figure 3:**
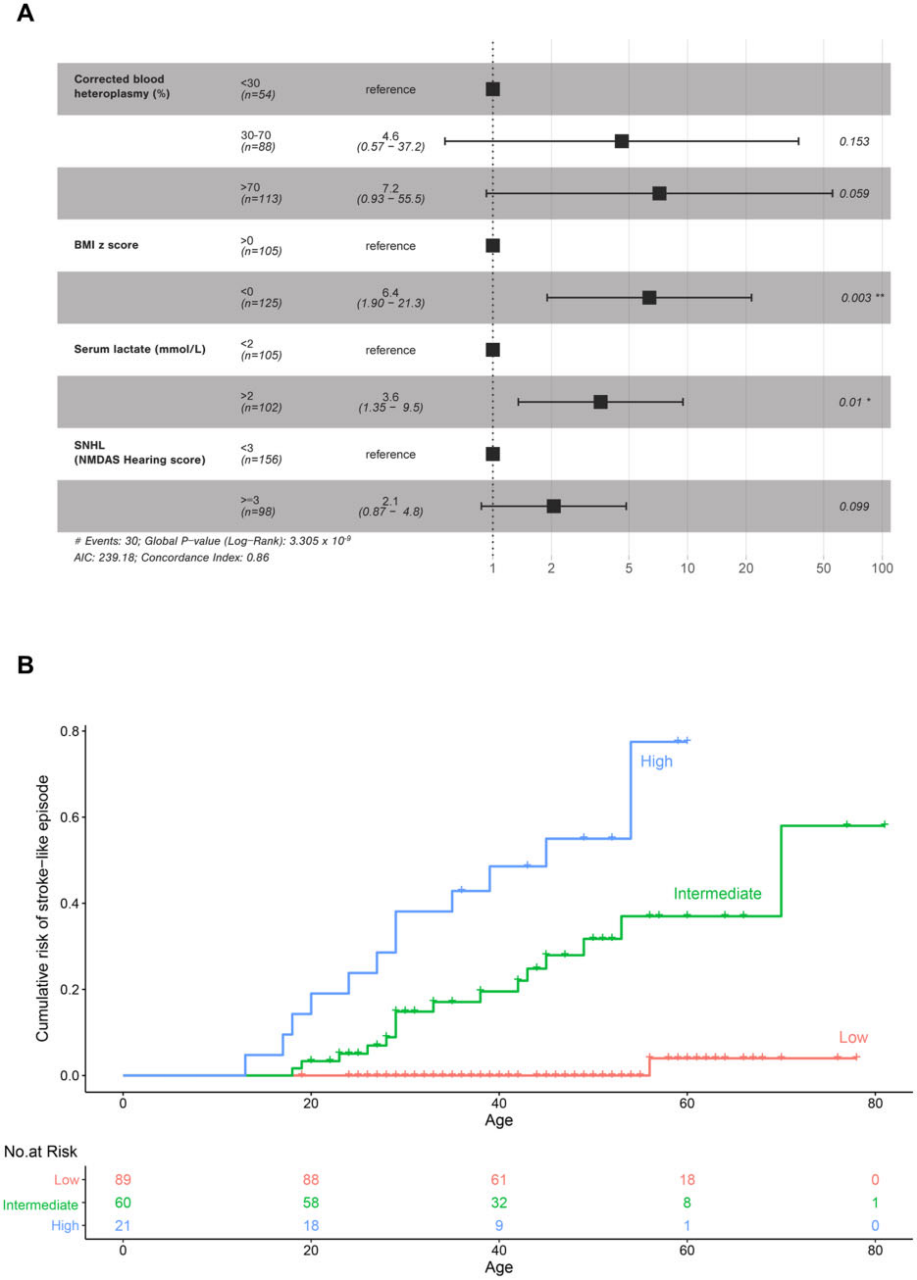
**Multivariate analyses of predictors for stroke-like episodes among individuals harbouring the m.3243A>G variant.** (**A**) Forest plot of the odds ratios (with 95% CI) for the four risk predictors of stroke-like episodes among m.3243A>G carriers. Corrected blood m.3243A>G heteroplasmy is derived from the methods detailed elsewhere.^72^ (**B**) Cumulative incidence of stroke-like episodes among the carriers of m.3243A>G variant according to their risk: high risk indicates the presence of all four risk predictors (6 points), intermediate the presence of three predictors (3–5 points), with low risk indicating between zero and two risk predictors (0–2 points). There are much fewer patients aged >60 years in our study, which may impact on the risk estimation.

Univariate analysis found significant associations with 11 putative predictors of stroke-like episodes (Supplementary Table 9). After simplification and model assessment using Akaike Information Criterion, a multivariate Cox proportional hazard analysis identified body mass index (BMI) *z*-score (HR 6.6, 95% CI 2.0–22.1, *P* = 0.003), corrected blood m.3243A>G heteroplasmy ≥70% (HR 7.2, 95% CI 0.9–55.5, *P* = 0.06), serum lactate > 2 mmol/l (HR 3.7, 95% CI 1.4–9.9, *P* = 0.01) and NMDAS hearing subscore ≥3 (indicative of sensorineural hearing loss severity; HR 2.0, 95% CI 0.8–4.74, *P* = 0.1) as four risk predictors of stroke-like episodes among m.3243A>G carriers (Fig. 3A, Supplementary Figs 4 and 5 and Supplementary Table 9). To account for sample size and enable clinical application (*n* = 170), a three-level hierarchical construct was defined which identified those patients at low (*n* = 89; 52%), intermediate (*n* = 60; 35%) and high risk (*n* = 21; 12%) of stroke-like episodes throughout their lifetime (Fig. 3B). This has allowed us to devise a 6-point prediction model for the absolute risk of developing a stroke-like episode in m.3243A>G carriers (high risk: 6 points; intermediate risk: 3–5 points; low risk: <2 points) with good overall discrimination (area under the curve: 0.87, 95% CI 0.82–0.93; *c*-statistics: 0.89; Supplementary Fig. 6).

The time interval between two consecutive stroke-like episodes was recorded in 65 patients, median 1.33 year (Q1 = 0.34, Q3 = 3.2, IQR = 2.86); there was no statistically significant difference in median interval time between events among mtDNA and *POLG* variants. An Andersen–Gill model of ordered multiple events demonstrated no significant differences in the baseline cumulative hazard for subsequent stroke-like episodes following the occurrence of a first event, suggesting that while stroke-like episodes are paroxysmal clinical events, the reoccurrence of an episode is independent of the initial event (Supplementary Figs 7 and 8).

### Neuropathological findings

#### Reduced brain weights and focal cortical necrosis

**Figure 4:**
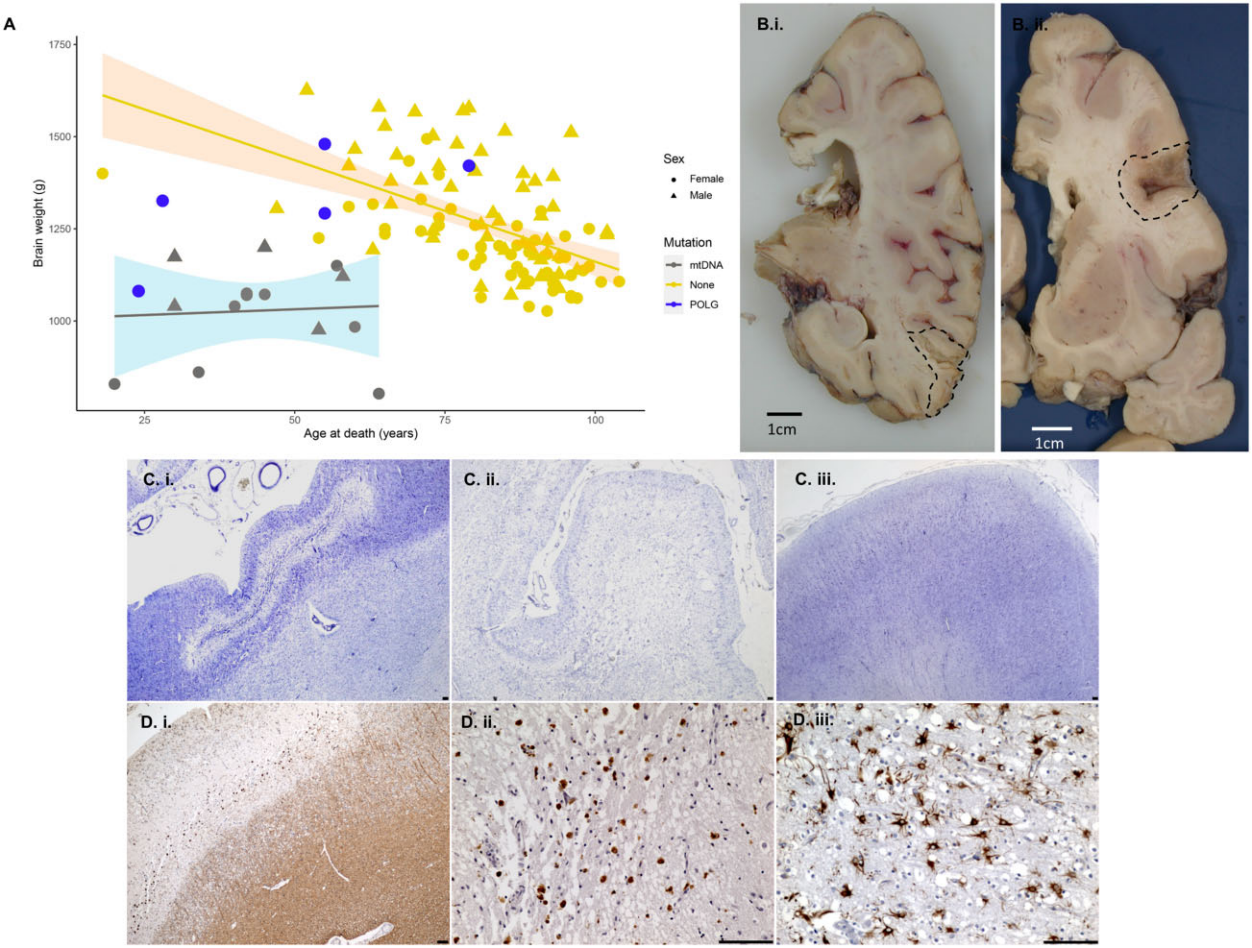
**Reduced brain weight and focal cortical necrosis are prominent across all genotypes**. Post-mortem brain weights (g) were significantly lower in patients with primary mtDNA disease relative to those harbouring *POLG* variants and control individuals (*P* = 0.005), particularly in those harbouring mtDNA variants (**A**). Macroscopic necrotic cortical lesions were evident in temporal lobe of Patient 3 [**B**(**i**); m.3243A>G] and frontal cortex (BA9) of Patient 14 [**B**(**ii**); *POLG*]. Scale bars = 1 cm. Stages of lesions in the temporal cortex range from selective laminar dehiscence [**C**(i); cresyl fast violet stain, CFV] to total necrosis of the temporal cortex in Patient 3 [**C**(**ii**); CFV] relative to normal cortex in a control [**C**(**iii**); CFV]. Evidence of intact myelination under the cortex of laminar dehiscence with myelin deposits in the lesioned grey matter in Patient 3 [**D**(**i**); myelin basic protein], these deposits also label with macrophages which may be phagocytosing damaged myelin [**D**(**ii**); CD-68]. Astrogliosis is observed circumferential to necrotic lesions in Patient 3 [**D**(**iii**); GFAP]. Scale bars = 100 µm.

Neuropathological findings of 26 patients (16 with documented ante-mortem stroke-like episodes) are summarized in Supplementary Table 10. Brain weights of patients affected by primary mtDNA disease were significantly decreased compared to those with recessive *POLG* variants and controls (*P* = 0.005; Fig. 4A). Macroscopic necrotic cortical lesions were evident upon brain dissection [Fig. 4B(i and ii)] with neuronal cell loss, ranging from selective neuronal dropout [Fig. 4C(i)] to laminar necrosis [Fig. 4C(ii)], a common neuropathological finding identified across all genotypes. This was associated with myelin deposits localized within necrotic foci [Fig. 4D(i)], which were immune-positive for phagocytosing macrophages [Fig. 4D(ii)] while underlying white matter remained myelinated. Astrogliosis is increased within necrotic foci [Fig. 4D(iii)]. The brain regions most affected by laminar necrosis included cerebellum (23/25; 92%), brainstem (16/24; 67%), temporal (14/25; 56%) and occipital lobes (13/25; 52%). Frontal and parietal lobes appeared less affected (7/24; 29%; Supplementary Table 11).

#### Respiratory chain deficiencies affecting the microvasculature

**Figure 5:**
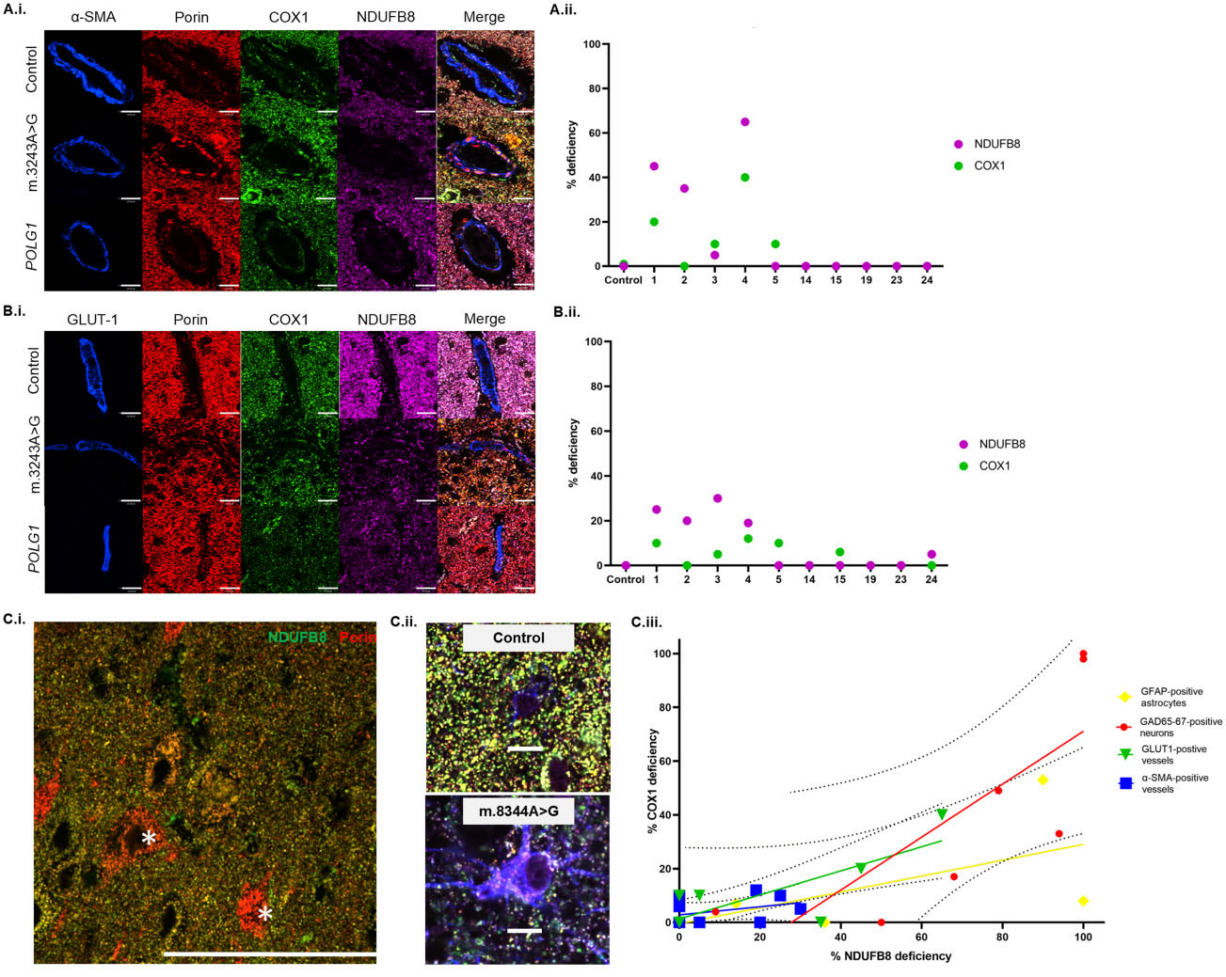
**Immunofluorescence showed mitochondrial OXPHOS deficiencies evident in microvessels, neurons and astrocytes in the occipital cortex**. Control arterioles (α-SMA; blue) demonstrated matched protein expression of OXPHOS subunits for complexes I (NDUFB8; magenta) and IV (COX1; green) relative to mitochondrial mass [porin; red; **A**(**i**)]. Arterioles in patients with primary mtDNA disease featured atypical ‘clumping’ of mitochondria [**A**(**i**)] and downregulation of NDUFB8 (magenta) and COX1 (green) proteins relative to porin (red) and therefore a higher percentage level deficiency [**A**(**ii**)], while no such alterations were observed in *POLG* patients. Control capillaries (GLUT-1; blue) have lower mitochondrial mass compared to the neuropil and do not show loss of OXPHOS subunit expression for complexes I (NDUFB8; magenta) and IV (COX1; green) relative to mitochondrial mass [porin; red; **B**(**i**)]. Capillaries in patients demonstrated decreased NDUFB8 (magenta) and COX1 (green) expression relative to porin [red; **B**(**i**)]. Quantification showed a higher percentage of deficiency, particularly in patients with primary mtDNA disease [**B**(**ii**)]. Scale bars = 14 μm. Immunofluorescent labelling of NDUFB8 (green) and porin (red) proteins showed neurons contain high mitochondrial mass with clear deficiencies of complex I [Patient 23; *POLG1*; **C**(**i**); complex I-deficient neurons shown by asterisk]. Scale bar = 100 µm. Immunofluorescent labelling of astrocytes [**C**(**ii**); GFAP = blue] and their mitochondria (magenta), NDUFB8 (red) and COXI (green) in patient and controls show reduced OXPHOS expression. Scale bar = 10 µm. Quantification of NDUFB8 and COXI within inhibitory interneurons [**C**(**iii**); GAD65–67-positive neurons; red] and astrocytes (GFAP-positive astrocytes; yellow) reveals high percentage levels of deficiency compared to microvessels [capillaries (GLUT1) green; and arterioles (α-SMA) blue].

Immunofluorescent analysis demonstrated that arterioles and capillaries have a low density of mitochondria (as judged by porin; Fig. 5A and B). Quadruple immunofluorescence showed reduced expression of OXPHOS subunits, complexes I and IV, relative to mitochondrial mass in microvessels from patients with primary mtDNA disease (Fig. 5A and B). In smooth muscle, mitochondrial mass was increased and demonstrated an atypical clumped appearance in patients who harboured pathogenic mtDNA variants (including m.3243A>G variant), suggestive of mitochondrial aggregation that was not observed in control tissues [Fig. 5A(i)]. Quantification confirmed the endothelium harboured higher levels of respiratory chain deficiency than smooth muscle [Fig. 5A(ii) and B(ii)]. Furthermore, the severity of respiratory chain dysfunction differed across different brain regions within the same patient, although this was particularly apparent in patients harbouring the m.3243A>G variant. Among this patient group, a hierarchy of regional dysfunction was observed with occipital cortex more severely affected than cerebellum, which was more severely affected than temporal cortex. However, the severity of respiratory chain defect did not correlate with the number of necrotic lesions (Supplementary Table 12).

#### Upregulation of SDHA protein and very mild reduction of COX1 protein in microvessels

Triple immunofluorescence confirmed that SDHA (succinate dehydrogenase complex, subunit A) protein expression was upregulated in patient vessels compared to porin and COX1 protein expression levels [Supplementary Fig. 9A(i)]. Quantification confirmed that *z*-scores were either within normal range or extremely high in patients’ vessels [Supplementary Fig. 9A(ii)].

#### Neurons versus astrocytes versus microvessels

Immunofluorescent labelling of NDUFB8 and porin within neurons revealed high mitochondrial mass [as judged by porin; Fig. 5C(i)] but variable levels of complex I subunit NDUFB8. Similar immunofluorescent labelling of astrocytes showed reduced levels of NDUFB8 and COXI in patients compared with controls [Fig. 5C(ii)]. To determine the extent of OXPHOS deficiency in neurons, astrocytes and microvessels, we used previously published data showing OXPHOS subunit expression in GAD65–67-positive interneurons and glial fibrillary acidic protein (GFAP)-positive astrocytes in the same cohort of patients.^38^ This analysis showed that neurons harbour greater percentage levels of OXPHOS deficiency relative to capillaries and arterioles [Fig. 5C(ii)].

## Discussion

Using a national cohort study and following well-established recommendations,^39^ we have developed a novel prognostic instrument to predict the risk of stroke-like episodes in individuals harbouring the pathogenic m.3243A>G variant. The multivariate model incorporates four items (BMI, blood lactate, age-corrected blood mtDNA heteroplasmy and sensorineural hearing loss) routinely available and easily applicable in clinical practice.^40^ Previous studies identified that patients with m.3243A>G-related MELAS have short stature and lower BMI,^41,42^ higher mtDNA heteroplasmy^43–45^ and serum lactate levels^45,46^ in addition to more severe hearing loss^45^ compared to m.3243A>G carriers not manifesting with stroke-like episodes (non-MELAS). However, these studies only reported the differences in various parameters between MELAS and non-MELAS patients as singular entities, as opposed to the quantification of risk of their cumulative impact as we have attempted here. The availability of a simple tool to predict and stratify future risk of MELAS syndrome will not only identify high-risk individuals and potentially enhance preventative strategies, but better inform patient counselling regarding their likely prognosis. Several stroke prediction models have been developed in disparate populations,^36^ but our prediction model demonstrates good overall discrimination and may pragmatically identify individuals with m.3243A>G-related mitochondrial disease at high risk of developing stroke-like episodes. These results, however, are only applicable in m.3243A>G-related mitochondrial disease, with other validated models widely recognized for other mechanistic forms of stroke.^36^

Since the initial 2012 report,^47^ our study suggests the estimated minimum prevalence of MELAS syndrome is higher at approximately 0.42 per 100 000. Study design, genetic background and population structure may,^47^ in part, explain this variability in prevalence. However, it is most likely due to improved awareness and case ascertainment.^48^

Almost one-third of all m.3243A>G cases had their first stroke-like episode after the age of 40 years; this is in contrast with the original diagnostic criteria for this syndrome.^49^ Individuals harbouring *POLG* variants sustained their first stroke-like episode earlier in life (second decade versus fourth decade) and had a more aggressive disease trajectory with a higher risk of death from status epilepticus^50^ compared to patients with mtDNA-related MELAS syndrome, who manifest with a more chronic disease course and died from predominantly non-CNS complications as reported elsewhere.^51,52^ Our cohort has a greater number of females harbouring pathogenic *POLG* recessive variants presenting with stroke-like episodes in their teens or early twenties, consistent with previous observations.^16,50^ It is intriguing that *POLG* disease comprises a continuum of clinical features including neonatal/infantile myocerebrohepatopathy spectrum, childhood/teenage-onset Alpers syndrome, teenage-/young adult-onset ataxia neuropathy spectrum, myoclonic epilepsy, myopathy and sensory ataxia, adult-onset chronic progressive external ophthalmoplegia, with the phenotypic manifestations clustering with age.^50,53^ Indeed, our clinical experience (unpublished) would corroborate previous findings that patients with late-onset disease (>40 years) predominantly present with chronic progressive external ophthalmoplegia, neuronopathy and cerebellar ataxia, while refractory epilepsy and stroke-like episodes are conspicuously rare.^16,50^

Approximately half of all *POLG* cases had an explosive onset of overt clinical seizures and were otherwise asymptomatic, outside of CNS features, prior to first MELAS syndrome presentation. This was in contrast to m.3243A>G patients in whom preceding systemic features were almost universal. These manifestations should serve as ‘red flag’ symptoms for the potential development of stroke-like episodes in m.3243A>G-related mitochondrial disease. Ictal epileptic headaches, recorded in three-quarters of individuals, often heralded the inception of a stroke-like episode, with 75% also exhibiting overt clinical seizure activity.^54^ Ictal epileptic headaches were often accompanied by a crescendo of elementary visual hallucinations and blindness, consistent with occipital lobe predilection, irrespective of genotype.^55,56^ Visual symptoms were almost invariably overlooked until more obvious clinical seizure activity, such as focal and/or generalized seizures, drowsiness and apathy of encephalopathy, ensued. This is likely to reflect the phenomenon of ictal spread,^57^ where subsequent epileptiform involvement of adjacent brain regions leads to clinical overshadowing of the original ictal focus. Extra-occipital manifestations including temporal lobe seizure phenomena (encompassing autonomic and psychic peculiarities) and receptive or expressive speech deficits also arose. Limb focal motor deficits and limb apraxia were incessantly subtle and often transient in nature. Detailed interrogation of seizure semiology often helped direct interpretation of clinical tests, including localization of the epileptogenic zone (particularly in cases of MRI-negative focal epilepsy), predicted disease trajectory^58^ and in rare cases advocated the use of novel interventions, as reported elsewhere.^59^

MRI abnormalities related to stroke-like episodes involved both cortical and subcortical areas and demonstrated variable lesion reversibility, characteristic of seizure-induced signal changes on MRI.^60–63^ Radiological evidence of cross-cerebellar diaschisis, the presence of the pulvinar sign^63^ and brain atrophy^64^ were indicative of prolonged seizure activity and favoured a particular pattern of seizure spread, including involvement of cortico-ponto-cerebellar pathways.^65^

Our findings support core descriptions of relapsing and remitting stroke-like episodes, both clinically and radiologically, and for the first time, we have systematically investigated the interval between stroke-like episodes. We have demonstrated that the median time interval of a second stroke-like episode was 1.33 year, and 25% of patients only experienced their second episode 3.2 years after the initial event. These findings highlight that the trajectory of stroke-like episodes is heterogeneous with important clinical implications. Clinicians, patients and their caregivers should be made readily aware of the nature of ictal phenomenology and risk of recurrent stroke-like episodes to ensure instigation of appropriate anticonvulsants. Moreover, the follow-up duration of any future trial of disease-modifying treatment for stroke-like episodes cannot be limited to ≤12 months based on the observation that half of the patients with MELAS syndrome only experience their second stroke-like episode 15 months after the first presentation.

Our neuroanatomical study represents the largest cohort reporting clinical and pathological correlates of stroke-like episodes in patients with mitochondrial disease. Post-mortem brain weights demonstrated marked reductions, particularly in patients harbouring pathogenic mtDNA variants versus patient with *POLG*-related disease, corroborating the disparity in tissue diminution observed on neuroimaging in our patient cohort. It is tempting to speculate that a more marked loss of brain weight in patients with mtDNA variants reflects their typically insidious onset and protracted course of disease, while patients with *POLG*-related MELAS have an explosive onset and rapid progression. Surprisingly, macroscopic neuropathological analysis of focal necrotic lesions was relatively innocuous compared to the striking nature of their neuroimaging alterations. Microscopically, they feature selective neuronal dropout to morphological changes of neurodegeneration, pan-cortical necrosis, astrogliosis,^66^ presence of microglial cells, blood brain barrier breakdown and secondary axonal loss, which are now recognized pathological hallmarks of various forms of medically refractory focal epilepsy,^66,67^ which were homologous across genotypes.

While some of our findings are confirmatory of previous studies that stroke-like episodes are not primarily driven by a vascular mechanism, we have shown for the first time that neurons harbour more severe mitochondrial respiratory chain deficits than vessels. Furthermore, our neuropathological data do not support a causal role of microvascular impairments driving the formation of focal necrotic cortical lesions. Mitochondrial respiratory chain deficiencies, involving complexes I and IV, were evident and detected at low levels within endothelial and smooth muscle cells of microvessels, particularly in patients harbouring pathogenic mtDNA variants. This contrasts to previous COX/SDH histochemistry data that demonstrated overt COX-deficiency within the microvasculature.^28,68^ Our data support increased SDHA protein levels within vessels that is not matched by either COX1 or porin protein expression; the relevance of a selective increase in SDH activity and expression in microvessels is not understood, but does explain the apparent high levels of COX deficiency. To measure the contribution of angiopathic changes to neuronal necrosis, we compared mitochondrial respiratory chain deficiencies within microvessels to the levels detected in inhibitory interneuron and astrocytic populations. This provided clear evidence that the neuronal deficit is more pronounced, and given the preponderance of inhibitory interneuron involvement^30^ provides further evidential support that these paroxysmal stroke-like events are seizure-induced and are not driven by angiopathic changes. Whether these paroxysmal events result from concomitant dysfunctional neuroinflammatory response to various triggers involving the interaction of neuronal, vascular and innate immune processes,^69^ as in other refractory seizure disorders, that is ultimately predicated on mitochondrial dysfunction^70^ remains to be determined.

There are several potential sources of bias identified including the retrospective nature of study design and cohort, variable data acquisition, inherent assumptions made for the development of the Cox model, limitation of surface EEG ability to detect deep lying seizure foci, potential sampling errors with the measurement of serum lactate^71^ and the lack of external validation of our prediction model. While other tissue heteroplasmy levels (including muscle) were available, numbers were insufficient and precluded their use in the model. Another potential limitation of the m.3243A>G-prediction model relates to ‘age’, which may represent yet another factor in prognostication. The age profile of first stroke-like episode is significantly different between m.3243A>G- and *POLG*-related cases. Among 72 patients who had m.3243A>G-related stroke-like episodes in our study, only four patients developed their first stroke-like episode before the age of 18 years. Our findings and clinical experience would suggest that m.3243A>G-related stroke-like episodes are uncommon in the paediatric population. The cumulative risk of developing stroke-like episodes for individuals harbouring the m.3243A>G variant may evolve over time, depending on the number of risk factors (as illustrated in Fig. 3B). However, the cumulative risk barely changes over time if an individual scores two or less in our prediction model, suggesting age is not an independent risk predictor.

Given no murine model has yet been developed that fully recapitulates the complex heterogeneity and pathogenesis of MELAS syndrome, the chronological analysis of pervasive stereotypical clinical features, neuroimaging alterations, EEG abnormalities and neuropathological findings to provide clarity around mechanisms underlying stroke-like episodes pathogenesis and physiology is imperative in our quest for curative therapies, and were strongly supportive of pharmacological refractory seizures driving the clinico-radiopathological correlates of stroke-like episodes and neurodegeneration across genotypes. In addition, we have pioneered the development of a tool to predict those individuals with genetically defined m.3243A>G-related mitochondrial disease, who are at high risk of developing stroke-like episodes and who may benefit from appropriate early clinical intervention. Our findings support the urgent need to revise current patient care standards, prioritizing instigation of anticonvulsant therapy early in the treatment strategy.^7^

## Supplementary Material

awab353_Supplementary_Data

## Abbreviations

MELAS: mitochondrial encephalopathy, lactic acidosis, and stroke-like episodes
OXPHOS: oxidative phosphorylation
