# Supplementary material for: Forecasting stroke-like episodes and outcomes in mitochondrial disease
Source: Brain. 2021 Dec 20;145(2):542–54. doi: 10.1093/brain/awab353 (PMC9014738; doi:10.1093/brain/awab353)
Supplement: awab353_Supplementary_Data [file awab353_supplementary_data.zip › brain-2021-00937-File009.pdf]

List of supplemental tables

Supplemental Table 1. Demographic summary of controls included for the study of vascular pathology.

Supplemental Table 2. Primary and secondary antibodies used in this study along with their optimised working conditions.

Supplemental Table 3. Catalogue of positive visual phenomena and visual field loss (confrontational assessment) in patients experiencing stroke-like episodes (n=56). B/L= bilateral. C= cerebellum. DWI= diffusion weighted imaging. F= frontal. I= insular. (L)= left. N/A= not available. P= parietal. O=occipital. T=temporal. Th= thalamus. (R)= right.

Supplemental Table 4. Mean age of last follow up for patients recruited to the UK Mitochondrial Disease Patient Cohort. CI= confidence interval. N/A= not applicable. SD= standard deviation. SLE= stroke-like episodes.

Supplemental Table 5. Comparison of disease burden of mitochondrial disease (measured by the mean total NMDAS score) between different groups. Data for the patients without stroke-like episodes were derived from the UK Mitochondrial Disease Patient Cohort. The number of subjects for individual categories are different from Supplemental Table 4 as not all patients had NMDAS completed. CI= confidence interval. SD= standard deviation. SLE= stroke-like episodes.

Supplemental Table 6. Comparison of neuroimaging changes between stroke-like episodes with and without status epilepticus. *Status epilepticus includes both prolonged focal and generalised seizures. p values were adjusted for multiple comparisons using the Benjamini-Hochberg method. ADC= apparent diffusion coefficient. PLED= periodic lateralising epileptic discharge. SLL= stroke-like lesion

Supplemental Table7. Summary of EEG findings. EEG data are available for 50 patients; m.3243A>G (n=30), other mtDNA mutations (n=7) and *POLG* mutations (n=13). EPC= epilepsia partialis continua. PLEDs = Periodic lateralized epileptiform discharges. * Significant difference in proportion between two groups

Supplemental Table 8. Relative Risk of death by 10 year age groupings of m.3243A>G patients. N/A= not available. SLE= stroke-like episode.

Supplemental Table 9. Univariate and multivariate analyses of putative factors associated with stroke-like episodes in the m.3243A>G group. NMDAS traits including threshold values for analysis as binary traits are based on the methods described elsewhere.[1] aHR = adjusted hazard ratio. CPEO= chronic progressive external ophthalmoplegia. eGFR= estimated glomerular filtration rate. HR= hazard ratio. NMDAS= Newcastle Mitochondrial Disease Adult Scale. *There are different upper normal limits between males and females for the following variables: haemoglobin, creatinine and CK level. ** The calculation of NMDAS cognition score is derived from three cognitive tests, namely The Wechsler Test of Adult Reading, the Symbol Search and Speed of Comprehension Test.

Supplemental Table 10. Summary of the cause of death and neuropathological findings (n=26). *These data have not been previously reported. Patients 1-17 had documented antemortem stroke-like episodes. Patients 18-26 did not have documented antemortem stroke-like episodes. BG = basal ganglia. CPEO= chronic progressive external ophthalmoplegia. ION= inferior olivary nucleus. KSS= Kearns-Sayre syndrome. MELAS= mitochondrial encephalomyopathy, stroke-like episodes and lactic acidosis. MERRF= myoclonic epilepsy and ragged red fibres. NA= not available. n.d.= not determined.

Supplemental Table 11. Comparison of the autopsy findings between mtDNA and *POLG* cases. [a]m.3243A>G (n=11), m.8344A>G (n=2), m.10010T>C (n=1), m.13094T>C (n=1), m.14685T>C (n=1), m.14709T>C (n=1), single, large-scale mtDNA deletion (n=1), *POLG* (n=8). [b]p values were adjusted for multiple comparisons.

Supplementary Table 12. Percentage level respiratory chain deficiency (NDUFB8 or COX1) in arterioles (α-SMA) or capillaries (GLUT1) throughout the occipital and temporal cortices and cerebellum and presence of focal necrotic lesion.

List of supplemental figures

Supplemental Figure 1. Classical changes of stroke-like lesions identified on CT head of m.3243A>G. (A) Symmetrical calcification in basal ganglia and thalami. (B) Low attenuation changes in the left occipital lobe. (C) Bilateral stroke-like lesions involving the occipital lobes, and these changes confirmed by restricted diffusion on MRI head. (D.i) A small area of low attenuation was present in the left temporal-occipital area, and the low attenuation became more confluent within two weeks (D.ii); gliotic changes identified in the same area with a significant focal atrophy (with dilated left lateral ventricle) 9 months later (D.iii). (E.i) Subtle hypo-attenuation in the left temporal pole. (E.ii) MRI head performed on the same day identified subacute changes involving bilateral temporal lobes, highlighting the limitation of CT head on identifying smaller lesions.

Supplemental Figure 2. Clinical features associated with stroke-like episodes.

Supplemental Figure 3. Serial MRI head scans of a 30 year old woman who has m.3243A>G-related MELAS syndrome. Her first stroke-like episode involved the right occipital lobe (A.i-ii) at the age of 20 years, and subsequent stroke-like episodes involved the left occipital lobe (B.i-ii), bilateral parietal lobes which were more marked on the right side (C.i-ii), right frontal lobe (D.i-ii) and most recently, the left front lobe (E.i-ii). Marked brain atrophy (evidenced by significant dilatation of ventricles) is now present (F.i-ii).

Supplemental Figure 4. Boxplots comparing non-MELAS and MELAS m.3243A>G patients in four parameters. Median, interquartile range (IQR), mean and 95% CI are provided in the bottom of each panel. (A) Body Mass Index (BMI) Z score. (B) Age-corrected blood m.3243A>G heteroplasmy.[2] (C) Serum lactate (normal <2.2 mmol/L). (D) Severity of sensorineural hearing loss (SNHL) based on NMDAS assessment (Question 2; Section 1). Patients with MELAS syndrome have significantly lower BMI, higher age-corrected blood heteroplasmy, higher serum lactate and more severe hearing loss than patients with non-MELAS syndrome ($p<0.001$).

Supplemental Figure 5. Univariate analysis of individual predictors for stroke-like episodes. (A) Corrected blood m.3243A>G heteroplasmy.[2] (B) BMI Z score denotes

the number of standard deviations above or below the reference mean derived from all patients with the m.3243A>G mutation. (C) Serum lactate level. (D) Severity of sensorineural hearing loss (SNHL) based on NMDAS assessment (Question 2; Section 1). HR= hazard ratio.

Supplemental Figure 6. Receiver operating characteristic (ROC) curve analysis of the predicted risk of developing stroke-like episodes in m.3243A>G carriers. The area under the curve (AUC) is 0.87 (95% CI 0.82-0.93) suggesting that the model combining four parameters (i.e. corrected blood m.3243A>G heteroplasmy, BMI Z score, serum lactate level and severity of SNHL) has good predictive ability to discriminate individuals with stroke-like episodes from other carriers of the m.3243A>G variant.[3]

Supplemental Figure 7. Clinical course of stroke-like episodes for individual patients. Red symbols = *POLG* cases, blue symbols = m.3243A>G cases and green symbols = other pathogenic mtDNA variants.

Supplemental Figure 8. Cumulative hazard for second, third, fourth and fifth stroke-like-episodes (Anderson-Gill Model). Pairwise comparisons were made between each using Log-Rank tests, with corrections for multiple comparisons by the Benjamini & Hochberg method.[4] All comparisons were non-significant ($p>0.05$), suggesting that while stroke-like episodes are paroxysmal phenomena, the reoccurrence of an episode is independent of the initial event. SLE = stroke-like episode.

Supplemental Figure 9. Immunofluorescent labelling of mitochondria (porin; red), complex II (SDHA; magenta), and complex IV (COX1; green) reveal a high level of SDHA expression relative to COX1 in patient microvessels from the occipital cortex (A. i.). Scale bar = 14 microns. Quantification of the SDHA optical densities and subsequent derivation of percentage expression levels confirm that all control microvessels demonstrate SDHA levels within a normal range (blue bar; A. ii.) while SDHA expression is either normal or increased in patient microvessels (blue or pink bars, respectively A. ii.).

Supplemental Table 1. Demographic summary of controls included for the study of vascular pathology.

Type	Age (years)	Sex	Length of fixation (weeks)	Post-mortem delay (hours)	Cause of death
**Control 1**	69	F	6	16	Gastric cancer
**Control 2**	68	M	8	54	Bowel cancer
**Control 3**	78	F	5	23	metastatic oesophageal carcinoma
**Control 4**	74	F	13	67	Lung cancer
**Control 5**	55	M	14	41	Liver cancer
**Control 6**	74	F	9	53	Heart failure and lung cancer
**Control 7**	70	M	11	72	Metastatic prostate cancer
**Control 8**	73	M	7	25	Not known
**Control 9**	78	F	8	34	Metastatic cancer
**Control 10**	45	M	13	13	Not known

Supplemental Table 2. Primary and secondary antibodies used in this study along with their optimised working conditions.

			**Immunohistochemistry**			
**Primary antibody**	**Host and Isotype**	**Source**	**Catalog No.**	**Antigen retrieval**	**Optimal dilution**	**Incubation conditions**
Collagen IV	Mouse monoclonal - IgG1	Sigma-Aldrich	C1926	N/A	1 in 500	1 hour, RT
			**Immunofluorescence**			
**Primary antibody**	**Host and Isotype**	**Source**	**Catalog No.**	**Antigen retrieval**	**Optimal dilution**	**Incubation conditions**
Glucose transporter 1 (GLUT-1)	Rabbit polyclonal - IgG	Thermo Scientific	PA1-21041	EDTA (1mM); 2100 antigen retriever	1 in 100	4°C, O/N
Alpha- Smooth muscle actin (α-SMA)	Rabbit polyclonal - IgG	Abcam	ab5694	EDTA (1mM); 2100 antigen retriever	1 in 100	4°C, O/N
Voltage-dependent anion channel 1 (VDAC1)	Mouse monoclonal - IgG2b	Abcam	ab14734	EDTA (1mM); 2100 antigen retriever	1 in 200	4°C, O/N
Complex I subunit NDUFB8	Mouse monoclonal - IgG1	Abcam	ab110242	EDTA (1mM); 2100 antigen retriever	1 in 100	4°C, O/N
Complex II subunit SDHA	Mouse monoclonal - IgG1	Abcam	ab14715	EDTA (1mM); 2100 antigen retriever	1 in 400	4°C, O/N
Complex IV subunit I	Mouse monoclonal - IgG2a	Abcam	ab14705	EDTA (1mM); 2100 antigen retriever	1 in 200	4°C, O/N
**Secondary antibody**	**Isotype**	**Source**	**Catalog No.**	**Antigen retrieval**	**Optimal dilution**	**Incubation conditions**
Biotin-SP conjugated	Goat Anti-Mouse IgG1 (γ1)	Jackson ImmunoResearch	115-065-205	N/A	1 in 100	2 hours, 4°C
Streptavidin, Alexa Fluor® 647 Conjugate	N/A	Life technologies	S32357	N/A	1 in 100	2 hours, 4°C
Alexa Fluor® 405	Goat Anti-Rabbit IgG (H+L)	Life technologies	A31556	N/A	1 in 100	2 hours, 4°C
Alexa Fluor® 488	Goat Anti-Mouse IgG2a (γ2a)	Life technologies	A21131	N/A	1 in 100	2 hours, 4°C
Alexa Fluor® 546	Goat Anti-Mouse IgG2b (γ2b)	Life technologies	A21143	N/A	1 in 100	2 hours, 4°C
Alexa Fluor® 647	Goat Anti-Mouse IgG1 (γ1)	Life technologies	A21240	N/A	1 in 100	2 hours, 4°C

Supplemental Table 3. Catalogue of positive visual phenomena and visual field loss (confrontational assessment) in patients experiencing stroke-like episodes (n=56).

Patient no	Genotype	Description of positive visual symptoms	Visual field loss	MRI changes
5	m.13094T>C	Flickering red circle in left visual field	Hazy left temporal field, left quadrantanopia	(L) O, T
7	m.13094T>C	Intermittent white flashing light evolved into constant presence in the left visual field for 2 days	Left visual field defect	(R) O
11	m.13513G>A	Double vision	Not known	N/A
13	m.3243A>G	Persistent shimmering in the left visual field for 3 months (equated to a moth)	Left heminanopia	N/A
14	m.3243A>G	Flashing light	Left heminanopia	(R) O, P
15	m.3243A>G	Flashing light	Not known	(B/L) O, P, T (DWI changes on right)
17	m.3243A>G	Colorful flashing light with pixel dots; flashing light, visual hallucination (insects) initially just unilateral then spread to the other eye	Right hemianopia (incomplete information on subsequent assessment)	(L) F, (B/L) I, (L) T, (B/L) O
18	m.3243A>G	Black & silver moving flashes - for 2 days	None	(L) O
20	m.3243A>G	Flashing light in the right visual field and visual hallucination	Not known	N/A
21	m.3243A>G	Flashing light for 4 days	Right hemianopia; developed cortical blindness few weeks later	(L) O
22	m.3243A>G	Bright coloured icons in the right visual field	Visual field defect	N/A
23	m.3243A>G	Flashing light; seeing worms creeping out from his skin; olfactory hallucination (offensive smell)	Left hemianopia evolving to cortical blindness	(R) O (DWI changes), (B/L) T
25	m.3243A>G	Visual hallucination	None	N/A
26	m.3243A>G	None	Visual field defect	(L) P, (B/L) T, (L) O
27	m.3243A>G	Flashing light in the right visual field	None	N/A
28	m.3243A>G	None	Hemianopia	(L) P, T
30	m.3243A>G	None	Right hemianopia	(B/L) F, I, T, P, O
31	m.3243A>G	Visual and auditory hallucination	Not known	N/A
32	m.3243A>G	Visual hallucination for weeks (even with eyes closed)	Left hemianopia	(R) I, O, P, T, Th, C
34	m.3243A>G	Colorful flashing light, pixel dots; auditory hallucination (fan noises);	Left upper quadrantanopia	(R) O
36	m.3243A>G	Flashing light for 2 days	Left visual loss (intermittent)	(R) O, P, T
37	m.3243A>G	No documented positive visual symptoms but reported auditory and olfactory hallucination, metallic taste for the first stroke-like episode; reported visual hallucination on second stroke-like episode (6 months later)	Left hemianopia	(R) P, T
38	m.3243A>G	Persistent flashing light in bilateral visual field	Variable visual defect	(R) O, P
39	m.3243A>G	Flashing light	Right hemianopia	(R) T
41	m.3243A>G	Coloured specked appearances for one week	Left hemianopia	(R) O, P, T, Th
44	m.3243A>G	Intermittent kaleidoscopic vision in left visual field	Left hemianopia	(R) P, (B/L) T (R>L)
45	m.3243A>G	Bright coloured icons in right visual field	Complete blind spot few days after the positive visual symptoms	(L) O, P
49	m.3243A>G	Intermittent kaleidoscopic vision in the right visual field, complex visual hallucination (human)	None	(R) P, (B/L) T, (B/L) O
51	m.3243A>G	Bright coloured objects in left visual field for 3 weeks	Left quadrantanopia	(R) O (DWI changes), (L) P, T
52	m.3243A>G	Floaters, flashing lights	Loss of colour vision	(B/L) O, T (DWI changes on the (R) O)
53	m.3243A>G	Colourful distorted shapes	None	(L) O, P, T
54	m.3243A>G	Black and white flashing lights in the upper left quadrant of visual field (present with eyes closed) for few months, which then evolved to coloured like kaleidoscope; flashing light and formed visual hallucination, and had difficulty with facial perception prosopagnosia	Visual agnosia and possibly optic ataxia; left hemianopia	(R) O, P, T

56	m.3243A>G	Coloured football/ Kaleidoscope	Right hemianopia	(R) F, I, P, T, O
57	m.3243A>G	Visual hallucination (birds across sky)	Visual field defect	(B/L) O
58	m.3243A>G	Flickery light in the right visual field, formed visual hallucination	Right hemianopia	(L) O, P
67	m.3243A>G	None	Cortical blindness	N/A
68	m.3243A>G	Not known	Left hemianopia	N/A
71	m.3243A>G	Yes	Visual loss	N/A
73	m.3243A>G	Flashing light	None	(B/L) T, (L) O
74	m.3243A>G	Not known	Left visual field loss	(B/L) T
75	m.3243A>G	Not known	Cortical blindness	(B/L) T, (R) O
76	m.5543T>C	Flashing light	Cortical blindness	(L) P, (L) T, (B/L) O
77	m.7541T>C	Visual hallucination	Visual field defect	(L) F, O, P, T
80	*POLG*	Rainbow-colored light in central vision formed visual hallucinations (animals)	Left hemianopia	(R) O, P
81	*POLG*	Difficulty distinguishing color (she believed a purple piece of card was shiny black); visual hallucination	Right visual field loss	(B/L) O, P, (R) Th
82	*POLG*	Visual hallucination (seeing ghost)	None	N/A
87	*POLG*	None	Left hemianopia	(B/L) O
88	*POLG*	None	Left hemianopia	(R) O
90	*POLG*	Flashing light	Quadrantopia	(R) O, P
92	*POLG*	Positive visual symptoms	No	(L) O, and Th, C
93	*POLG*	Formed visual hallucination, flashing light (present with eyes closed)	Blurred vision	(L) O, P and Th, C
94	*POLG*	None	Right hemianopia	(B/L) F, (L) I, P, T, O
95	*POLG*	Coloured flashing light	No	N/A
100	*POLG*	Not known	Central scotoma	(L) P, T, O
103	m.3243A>G	Visual aura, photophobia	Not known	N/A
109	m.3764C>G	Visual problems (visual field and visual acuity) and appeared spells with bilateral eyelid clonic movements, right side eyes deviation (duration 5-10 second, frequency 10-20 per day).	Right hemianopia	(L) P, (B/L) O (DWI changes on (R) O)

B/L= bilateral. C= cerebellum. DWI= diffusion weighted imaging. F= frontal. I= insular. (L)= left. N/A= not available. P= parietal. O=occipital. T=temporal. Th= thalamus. (R)= right.

Supplemental Table 4. Mean age of last follow up for patients recruited to the UK Mitochondrial Disease Patient Cohort.

		POLG			m.3243A>G	
			p value			p value
Mean age of last follow up (95% CI; SD)	Male (n=24)	46.2 (37.0-55.4; 21.7)	0.182	Male (n=138)	41.4 (39.1-43.8; 14.1)	0.009
	Female (n=43)	37.7 (30.5-45.0; 23.5)		Female (n=202)	46.0 (43.6-48.4; 17.2)	
	SLE (n=23)	22.3 (15.7-28.9; 5.3)	<0.001	SLE (n=72)	35.7 (30.7-40.8; 14.5)	<0.001
	No SLE (n=39)	56.6 (50.3-62.8;15.5)		No SLE (n=265)	46.8 (44.9-48.7; 14.4)	

CI= confidence interval. N/A= not applicable. SD= standard deviation. SLE= stroke-like episodes.

Supplemental Table 5. Comparison of disease burden of mitochondrial disease (measured by the mean total NMDAS score) between different groups.

Genotype	Subset	N	Mean NMDAS (95% CI)	SD	p value
**All data**	*POLG*	32	45.6 (38.0-53.3)	21.2	<0.0001
	m.3243A>G	251	22.9 (20.5-25.3)	19.5	
POLG	SLE	5	51.6 (31.1-72.2)	16.5	0.71
	No SLE	26	45.8 (37.2-54.4)	21.4	
**m.3243A>G**	SLE	34	50.7 (44.0-57.4)	19.2	<0.0001
	No SLE	217	18.6 (16.5-20.7)	15.6	
**All SLE**	*POLG*	5	51.6 (31.1-72.2)	16.5	0.98
	m.3243A>G	34	50.7 (44.0-57.4)	19.2	

Data for the patients without stroke-like episodes were derived from the UK Mitochondrial Disease Patient Cohort. The number of subjects for individual categories are different from Supplemental Table 4 as not all patients have had NMDAS completed. CI= confidence interval. SD= standard deviation. SLE= stroke-like episodes.

Supplemental Table 6. Comparison of neuroimaging changes between stroke-like episodes with and without status epilepticus.

	No status	Status epilepticus*	p value
**Genotype**			<0.001
mtDNA	68/69 (99%)	19/46 (41%)	
POLG	1/69 (1%)	27/46 (59%)	
**Neuroimaging changes**			
Thalamic	3/69 (4%)	12/46 (26%)	0.004
Cross-cerebellar	6/69 (9%)	12/46 (26%)	0.028
Frontal	13/69 (19%)	17/46 (37%)	0.053
Insular	13/69 (19%)	4/46 (9%)	0.156
Parietal	47/69 (68%)	25/46 (54%)	0.156
Temporal	51/69 (74%)	18/46 (39%)	0.004
Occipital	52/69 (75%)	29/46 (63%)	0.156
**Median no of SLL (range)**	3 (9)	3 (11)	0.508
**ADC map changes**			0.041
No change	2/50 (4%)	5/31 (16%)	
Increased	28/50 (56%)	11/31 (36%)	
Decreased	2/50 (4%)	5/31 (16%)	
Mixed	18/50 (36%)	10/31 (32%)	

*Status epilepticus includes both prolonged focal and generalised seizures. p values were adjusted for multiple comparisons using the Benjamini-Hochberg method. ADC= apparent diffusion coefficient. PLED= periodic lateralising epileptic discharge. SLL= stroke-like lesion

Supplemental Table 7. Summary of EEG findings.

	mtDNA	*POLG*	p value
**Number of patients with acute EEG**	27	11	-
**Number of acute EEG (median; range)**	120 (4; 1-16)	89 (6; 1-17)	-
**EEG changes**			
Encephalopathic changes	114/118 (97%)	65/83 (78%)	0.036
Burst suppression	0	9/83 (11%)	
Other	0	1	
Distribution of epileptic discharge			<0.001
None*	42/120 (35%)	20/89 (22%)	
Anterior	14/120 (12%)	11/89 (12%)	
Anterior and posterior	0	2/89 (2%)	
Temporal	9/120 (8%)	5/89 (6%)	
Central	4/120 (3%)	0	
Posterior*	23/120 (19%)	38/89 (43%)	
Posterior and temporal	4/120 (3%)	0	
Generalised	6/120 (5%)	0	
Unilateral, non-specified	6/120 (5%)	5/89 (6%)	
EPC without EEG correlate	0	6/89 (7%)	
No information	12/120 (10%)	2/89 (2%)	
**PLEDs**	7/35 (20%)	21/56 (38%)	
**Number of patients with routine EEG**	26	5	-
**Number of routine EEG (median; range)**	42 (1;1-4)	11 (2; 1-4)	-
**EEG changes**			
Encephalopathy changes	34/42 (81%)	10/11 (91%)	0.515
Epileptic discharge	21/42 (50%)	3/11 (27%)	0.173
Anterior	4/23 (17%)	0	
Anterior and posterior	0	0	
Temporal	10/23 (43%)	0	
Central	0	0	
Posterior	3/23 (13%)	3/3 (100%)	
Posterior and temporal	0	0	
Generalised	0	0	
Unilateral, non-specified	3/23 (13%)	0	

EEG data are available for 50 patients; m.3243A>G (n=30), other mtDNA mutations (n=7) and *POLG* mutations (n=13). EPC= epilepsia partialis continua. PLEDs = Periodic lateralized epileptiform discharges. * Significant difference in proportion between two groups

Supplemental Table 8. Relative Risk of death by 10 year age groupings of m.3243A>G patients.

	**No SLE**		**SLE**		**Relative Risk**
**Age Group**	No Death	Death	No Death	Death	
<10	2	0	0	0	N/A
10-19	13	1	6	3	6.5
20-29	27	1	12	5	11.3
30-39	51	2	6	4	17.0
40-49	46	3	8	8	15.3
50-59	54	3	8	8	18.0
60-69	36	5	2	2	7.2
70-79	14	0	0	1	N/A
80-89	1	1	0	0	N/A

N/A= not available. SLE= stroke-like episode.

Supplemental Table 9. Univariate and multivariate analyses of putative factors associated with stroke-like episodes in the m.3243A>G group.

Variable	Subset	Total	MELAS		Univariate analysis		Multivariate analysis		
			Yes	No	HR (95% CI)	p value	aHR (95% CI)	p value	AIC change
**Sex**	Male	121	22	99	2.3 (1.2-4.4)	**0.008**	N/A	0.66	49.1
	Female	183	18	165					
**Family History of stroke-like episodes (Binomial)**	1	131	13	118	0.6 (0.3-1.1)	0.091	-	-	-
	0	140	25	115					
**Blood Heteroplasmy (%)**	>70	113	25	88	8.6 (2.0-36.5)	**0.004**	7.2 (0.9-55.5)	0.06	-26.2
	30-70	88	9	79	4.0 (0.9-18.7)	0.077	4.59 (0.6-37.2)	0.153	
	<30	54	2	52					
**eGFR**	>60	84	12	72	0.9 (0.2-3.0)	0.808	-	-	-
	<=60	10	3	7					
**Haemoglobin (g/L)***	>130/115	220	28	192	0.5 (0.2-1.8)	0.129	-	-	-
	<=130/115	32	8	23					
**Creatinine (umol/L)***		27	6	21	1.8 (0.2-1.3)	0.179	-	-	-
		231	31	200					
**Serum lactate (mmol/L)**	>2	102	29	73	6.0 (2.5-14.4)	**<0.001**	3.7 (1.4-9.9)	0.01	-18.6
	<=2	105	6	99					
**Creatine kinase (CK) (U/L)***	>320/200	29	6	23	1.5 (0.6-3.7)	0.345	-	-	-
	<=320/200	190	26	164					
**BMI Z score**	<0	125	30	95	6.2 (2.4-16.1)	**<0.001**	6.6 (2.0-22.1)	0.003	-22.5
	>0	104	5	100					
**NMDAS Hearing**	3-5	98	26	72	3.1 (1.5-6.4)	**0.003**	2.0 (0.8-4.7)	0.1	-17.6
	0-2	156	10	146					
**NMDAS Diabetes**	2-5	105	19	86	1.1 (0.5-2.0)	0.877	-	-	-

	0-1	150	18	132					
**NMDAS Cardiovascular**	2-5	62	12	50	1.4 (0.7-2.8)	0.404	-	-	-
	0-1	168	21	147					
**NMDAS Gastro-Intestinal Disturbance**	4-5	14	4	10	2.3 (0.8-6.5)	0.117	-	-	-
	0-3	241	33	208					
**NMDAS Psychiatric involvement**	3-5	32	10	22	2.9 (1.4-6.0)	**0.004**	N/A	0.98	43.5
	0-2	223	27	196					
**NMDAS Ataxia**	2-5	82	25	57	3.7 (1.8-7.8)	**<0.001**	N/A	0.62	50.5
	0-1	170	10	160					
**NMDAS Migraine**	5	27	4	23	1.4 (0.5-4.0)	0.512	-	-	-
	0-4	227	32	195					
**NMDAS Dysphonia-Dysarthria**	2-5	28	18	10	8.5 (4.4-16.4)	**<0.001**	N/A	0.08	16.8
	0-1	225	18	207					
**NMDAS Visual Acuity**	3-5	8	4	4	4.4 (1.5-12.4)	**0.006**	N/A	0.97	45.5
	0-2	239	30	209					
**NMDAS Ptosis**	2-5	35	10	25	2.0 (1.0-4.2)	0.065	-	-	-
	0-1	218	26	192					
**NMDAS CPEO**	2-5	26	6	20	1.7 (0.7-4.0)	0.26	-	-	-
	0-1	226	29	197					
**NMDAS Myopathy**	2-5	70	17	53	2.2 (1.1-4.2)	**0.022**	N/A	0.84	47.4
	0-1	182	18	164					

NMDAS traits including threshold values for analysis as binary traits are based on the methods described elsewhere.[1] aHR = adjusted hazard ratio. CPEO= chronic progressive external ophthalmoplegia. eGFR= estimated glomerular filtration rate. HR= hazard ratio. NMDAS= Newcastle Mitochondrial Disease Adult Scale. *There are different upper normal limits between males and females for the following variables: haemoglobin, creatinine and CK level. ** The calculation of NMDAS cognition score is derived from three cognitive tests, namely The Wechsler Test of Adult Reading, the Symbol Search and Speed of Comprehension Test.

Supplemental Table 10. Summary of the patient clinical and neuropathological findings (n=26).

Patient (Sex)	Genotype	Age of Onset/ Death	Clinical features	Cause of death	Brain Weight*	Cortical necrotic lesions	Basal ganglia*	Cerebellum	Brainstem*
**1 (F)**	m.3243A>G	30/36	MELAS; epilepsy, cognitive impairment, ataxia, deafness, gut dysmotility, cardiomyopathy	Cardiac arrest	930g	Temporal, hippocampal CA1 and CA2, parietal and occipital	Striatal mineralisation	Purkinje, dentate nucleus and granule layer cell loss	Inferior olivary nucleus and dorsal motor nucleus of vagus nerve cell loss
**2 (F)**	m.3243A>G	51/60	MELAS; epilepsy, cognitive impairment, ataxia, deafness, diabetes, cardiomyopathy	Multi-organ failure	984g	Frontal and hippocampal CA1	Pallidal mineralisation	Purkinje and granule layer cell loss	Locus coeruleus and dorsal motor nucleus of vagus nerve cell loss
**3 (M)**	m.3243A>G	41/45	MELAS; epilepsy, cognitive impairment, ataxia	Mitochondrial disease	1200g	Frontal and temporal	Pallidal mineralisation	Focal necrotic lesion. Purkinje and dentate nucleus cell loss	Loss of substantia nigra neurons
**4 (F)**	m.3243A>G	10/20	MELAS; epilepsy, cognitive impairment, ataxia, deafness.	Aspiration pneumonia	829g	Frontal, temporal, insula, parietal and occipital	Striatal, pallidal and thalamic mineralisation; loss of pulvinar neurons	Focal necrotic lesion. Purkinje, dentate nucleus and granule layer cell loss	Mineralisation of cerebral peduncle vessels
**5 (M)**	m.8344A>G	57/58	MERRF/MELAS syndrome; epilepsy, cognitive impairment, ataxia, peripheral neuropathy	Stroke-like episodes	1121g	Temporal, parietal and occipital	Loss of striatal and pallidal neurons	Purkinje, dentate nucleus and granule layer cell loss	Neuronal loss in locus coeruleus, ION and cuneate nucleus
**6 (F)**	m.3243A>G	NA/42	MELAS; ataxia, diabetes, neuropathy	N/A	1070g	Temporal, hippocampal CA1, parietal, occipital	None	Purkinje, dentate nucleus and granule layer cell loss	Neuronal loss in dorsal motor nucleus of vagus nerve
**7 (F)**	m.3243A>G	40/57	MELAS; gut dysmotility, cardiomyopathy	Congestive heart failure	1150g	Temporal, occipital	Striatal mineralisation, lacunar state	Multiple focal necrotic lesions. Loss of Purkinje, dentate nucleus and granule layer neurons	Neuronal loss in inferior olivary nucleus
**8 (M)**	m.3243A>G	41/45	MELAS; cognitive impairment, gut dysmotility	Sepsis	1040g	Frontal, temporal, parietal, occipital	Pallidal mineralisation	Multiple focal necrotic lesions. Loss of Purkinje, dentate nucleus and granule layer neurons	Neuronal loss in locus coeruleus
**9 (F)**	m.3243A>G	45/64	MELAS; epilepsy, cognitive impairment, ataxia, deafness, diabetes	End-stage mitochondrial disease	803g	Temporal	Pallidal mineralisation	Purkinje cell loss	Pontine microinfarct
**10 (M)**	m.3243A>G	45/54	MELAS; epilepsy, cognitive impairment, ataxia, deafness, diabetes, gut dysmotility	Aspiration pneumonia	976g	Temporal, parietal, occipital	Pallidal mineralisation	Purkinje cell loss	None

**11 (M)**	m.3243A>G	33/54	MELAS; epilepsy, cognitive impairment, ataxia, deafness, diabetes, gut dysmotility	Aspiration pneumonia	861g	Hippocampal CA2 and CA3	Pallidal mineralisation	Dentate nucleus mineralisation	None
**12 (F)**	m.13094T>C	33/34	Leigh syndrome-MELAS overlap; epilepsy and ataxia	Respiratory failure (likely centrally mediated)	1260g	Temporal, occipital, insular	Lacunar state; loss of subthalamic nucleus neurons	Loss of Purkinje, dentate nucleus and granule layer neurons	Neuronal loss in superior colliculus and oral pontine reticular nucleus
**13 (F)**	m.10010T>C	21/51	Focal and generalised seizures, ataxia, extrapyramidal movement disorders, optic atrophy, deafness	Chest infection	884g	Occipital	Striatal mineralisation; loss of putamen neurons	Loss of Purkinje and granule layer neurons; dentate vessel mineralisation	None
**14 (M)**	*POLG* (ar)	NA/79	CPEO, ataxia, cognitive impairment	Pneumonia	1421g	Frontal and parietal	Pallidal lacunes	Purkinje cell loss	Loss of substantia nigra, dorsal motor nucleus of vagus nerve, locus coeruleus and inferior olivary nucleus neurons; alongside Lewy body pathology
**15 (F)**	*POLG* (ar)	20/24	Epilepsy, myoclonus, ataxia, cognitive impairment, CPEO	Suppurative tracheobronchitis	1081g	Occipital	None	Focal necrotic lesion. Purkinje and dentate nucleus cell loss	Not evaluated
**16 (F)**	*POLG* (ar)	16/28	Refractory epilepsy and ataxia	Status epilepticus	1326g	Occipital	None	Purkinje cell loss	None
**17 (F)**	*POLG* (ar)	18/23	Refractory epilepsy, liver failure with spontaneous recovery (after exposure to valproate), ataxia, neuropsychiatric symptoms	Status epilepticus	n.d.	Occipital	None	Purkinje cell loss	None
**18 (M)**	m.3243A>G	19/30	Hypertrophic cardiomyopathy, deafness	Heart failure secondary to end-stage hypertrophic cardiomyopathy	1174g	Not determined	Thalamus	Not determined	Neuronal loss in substantia nigra
**19 (F)**	m.8344A>G	18/42	MERRF syndrome with myoclonic epilepsy, cognitive impairment, ataxia, peripheral neuropathy	Respiratory failure	1075g	None	Striatal, pallidal and thalamic perivascular lymphoid cuffing	Focal necrotic lesion. Loss of Purkinje, dentate nucleus and granule layer neurons	Neuronal loss in substantia nigra, locus coeruleus, inferior olivary nucleus and cuneate nucleus
**20 (M)**	m.14709T>C	34/55	Cognitive impairment, peripheral neuropathy, diabetes, CPEO, ischaemic heart disease, severe peripheral vascular disease	Myocardiac infarction	1325g	Occipital, parietal, hippocampal CA2	None	Purkinje and dentate nucleus cell loss	None

**21 (F)**	m.14685T>C	7/45	Bilateral cataracts, retinitis pigmentosa, progressive paraparesis, cognitive impairment, ataxia, dysphagia, aphasia, deafness, lactic acidaemia	Pneumonia	1072g	Normal neuronal population; white matter changes	Cavitation and neuronal loss; striatal, pallidal and thalamic mineralisation	Normal	Neuronal loss in inferior olivary nucleus
**22 (F)**	Single mtDNA deletion	3/40	KSS, ataxia, dementia	Mitochondrial disease	1040g	White matter degeneration	Pallidal mineralisation	White matter loss; loss of Purkinje, dentate nucleus and granule layer neurons	None
**23 (M)**	*POLG* (ar)	22/59	Parkinsonism, CPEO, peripheral neuropathy, ataxia	Mitochondrial disease	1480g	Frontal and temporal	Striatal microinfarct	Focal necrotic lesion. Purkinje and dentate nucleus cell loss	Neuronal loss in substantia nigra and Lewy body pathology; neuronal loss in dorsal motor nucleus of vagus nerve
**24 (M)**	*POLG* (ar)	NA/55	Epilepsy, cognitive impairment, ataxia, myoclonus, CPEO, peripheral neuropathy, deafness, mild learning disability, tremor	Lower respiratory tract infection	1292g	Temporal	None	Focal necrotic lesion. Loss of Purkinje, dentate nucleus and granule layer neurons	Neuronal loss in substantia nigra, dorsal motor nucleus of vagus nerve, locus coeruleus and inferior olivary nucleus
**25 (M)**	*POLG* (ar)	15/50	Generalised tonic-clonic seizures, CPEO, ataxia and peripheral neuropathy	Suppurative tracheobronchitis	n.d.	Hippocampal sectors CA1-4	None	Purkinje and dentate nucleus cell loss	Neuronal loss in substantia nigra and inferior olivary nucleus
**26 (F)**	*POLG* (ar)	50/74	CPEO, ataxia, peripheral neuropathy	Aspiration pneumonia	1225g	None	Pallidal mineralisation	Purkinje cell loss	None

*These data have not been previously reported. Patients 1-17 had documented antemortem stroke-like episodes. Patients 18-26 did not have documented antemortem stroke-like episodes. BG = basal ganglia. CPEO= chronic progressive external ophthalmoplegia. ION= inferior olivary nucleus. KSS= Kearns-Sayre syndrome. MELAS= mitochondrial encephalomyopathy, stroke-like episodes and lactic acidosis. MERRF= myoclonic epilepsy and ragged red fibres. NA= not available. n.d.= not determined.

Supplemental Table 11. Comparison of the autopsy findings between mtDNA and *POLG* cases (n=26).

	**mtDNA**[a]	***POLG***	**p value**[b]
N	18	8	-
Age of death	44.4 +/- 13	44.9 +/- 20.8	0.955
Brain weight (g)	1044 +/- 148	1304 +/- 142	0.005
History of ante-mortem SLE	13/1	4/7	0.647
Location of Cortical lesion			
Frontal	5/17	2/7	0.957
Parietal	6/17	1/7	0.505
Temporal	12/17	2/8	0.075
Occipital	10/17	3/8	0.432
Cerebellum	15/17	8/8	1
Basal ganglia	15/17	3/8	0.056
Corpora amylacea	6/13	6/6	0.075

[a]m.3243A>G (n=11), m.8344A>G (n=2), m.10010T>C (n=1), m.13094T>C (n=1), m.14685T>C (n=1), m.14709T>C (n=1), single, large-scale mtDNA deletion (n=1), *POLG* (n=8)

[b]p values were adjusted for multiple comparisons.

Supplementary Table 12. Percentage level respiratory chain deficiency (NDUFB8 or COX1) in arterioles (α-SMA) or capillaries (GLUT1) throughout the occipital and temporal cortices and cerebellum and presence of focal necrotic lesion.

		**Occipital**					**Temporal**					**Cerebellum**				
		**α-SMA**		**GLUT1**		**Necrotic lesion**	**α-SMA**		**GLUT1**		**Necrotic lesion**	**α-SMA**		**GLUT1**		**Necrotic lesion**
**Genetic defect**	**No.**	NDUFB8	COX1	NDUFB8	COX1		NDUFB8	COX1	NDUFB8	COX1		NDUFB8	COX1	NDUFB8	COX1	
**Control**		0	0	0	1	-	1	0	0	1	-	0	2	0	0	-
**m.3243A>G**	1	25	10	45	20	Yes	5	0	5	0	Yes	25	20	25	15	
**m.3243A>G**	2	20	0	35	0		0	0	10	0	Yes	0	0	34	20	
**m.3243A>G**	3	30	5	5	10		0	0	0	0		10	0	5	0	Yes
**m.3243A>G**	4	19	12	65	40	Yes	13	19	38	14	Yes	10	10	50	67	Yes
**m.8344A>G**	5	0	10	0	10	Yes	0	5	0	0	Yes	0	0	0	0	Yes
**m.8344A>G**	19	0	0	0	0		0	0	0	0		0	0	0	0	Yes
POLG	14	0	0	0	0		0	0	0	0		0	0	0	0	
POLG	15	0	6	0	0	Yes	0	0	0	0		11	0	10	0	Yes
POLG	23	0	0	0	0		0	0	5	5	Yes	0	0	0	0	Yes
POLG	24	5	0	0	0		0	0	0	0	Yes	0	0	0	0	Yes

REFERENCES

1. Pickett SJ, Grady JP, Ng YS, et al. Phenotypic heterogeneity in m.3243A>G mitochondrial disease: The role of nuclear factors. *Annals of clinical and translational neurology*. Mar 2018;5(3):333-345. doi:10.1002/acn3.532

2. Grady JP, Pickett SJ, Ng YS, et al. mtDNA heteroplasmy level and copy number indicate disease burden in m.3243A>G mitochondrial disease. *EMBO Molecular Medicine*. 2018;10(6)doi:10.15252/emmm.201708262

3. Hanley JA, McNeil BJ. The meaning and use of the area under a receiver operating characteristic (ROC) curve. *Radiology*. Apr 1982;143(1):29-36. doi:10.1148/radiology.143.1.7063747

4. Benjamini Y, Hochberg Y. Controlling the False Discovery Rate: A Practical and Powerful Approach to Multiple Testing. *Journal of the Royal Statistical Society Series B (Methodological)*. 1995;57(1):289-300.
