# Supplementary material for: Forecasting stroke-like episodes and outcomes in mitochondrial disease
Source: Brain. 2021 Dec 20;145(2):542–54. doi: 10.1093/brain/awab353 (PMC9014738; doi:10.1093/brain/awab353)

Supplemental Figure 1

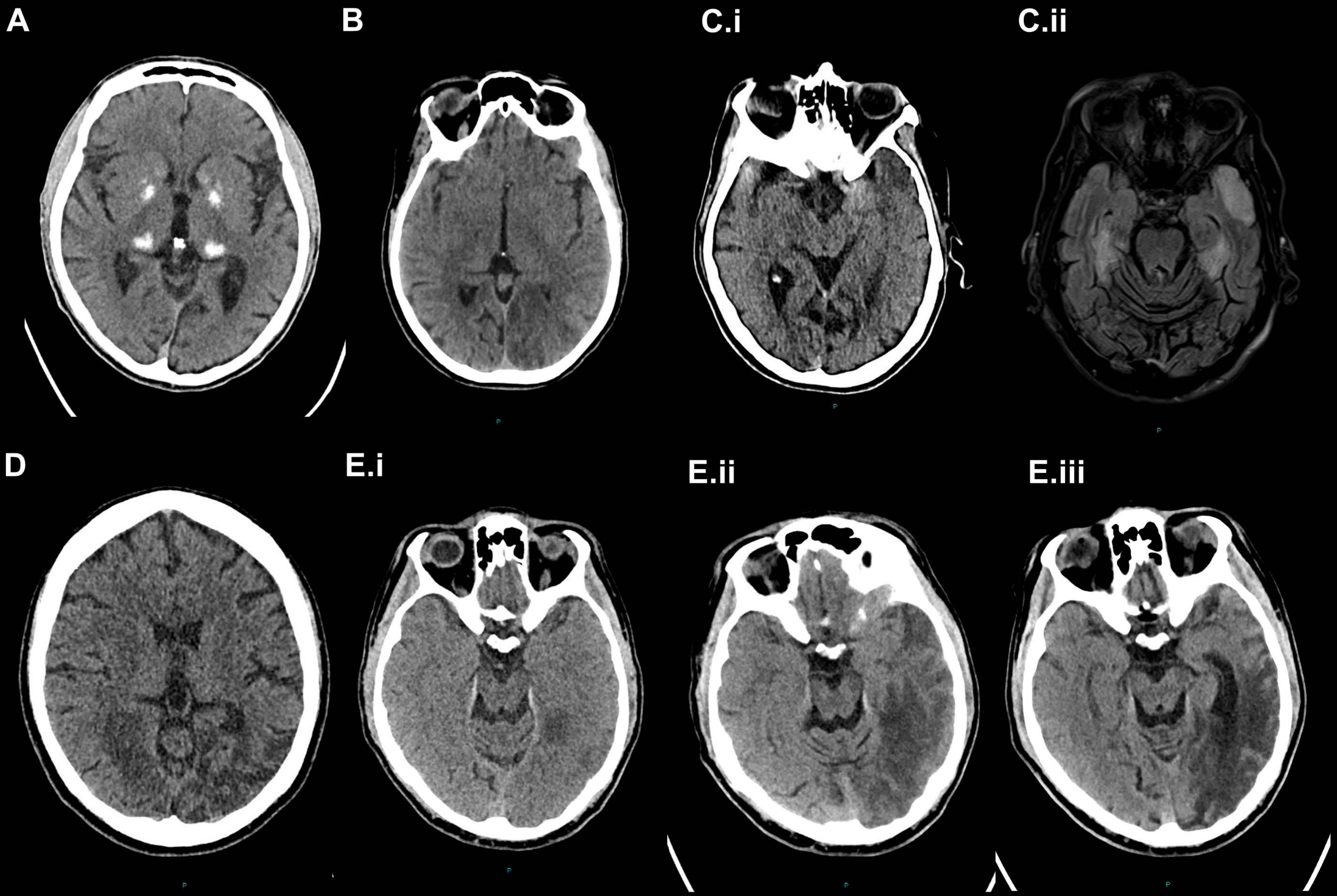

Supplemental Figure 2

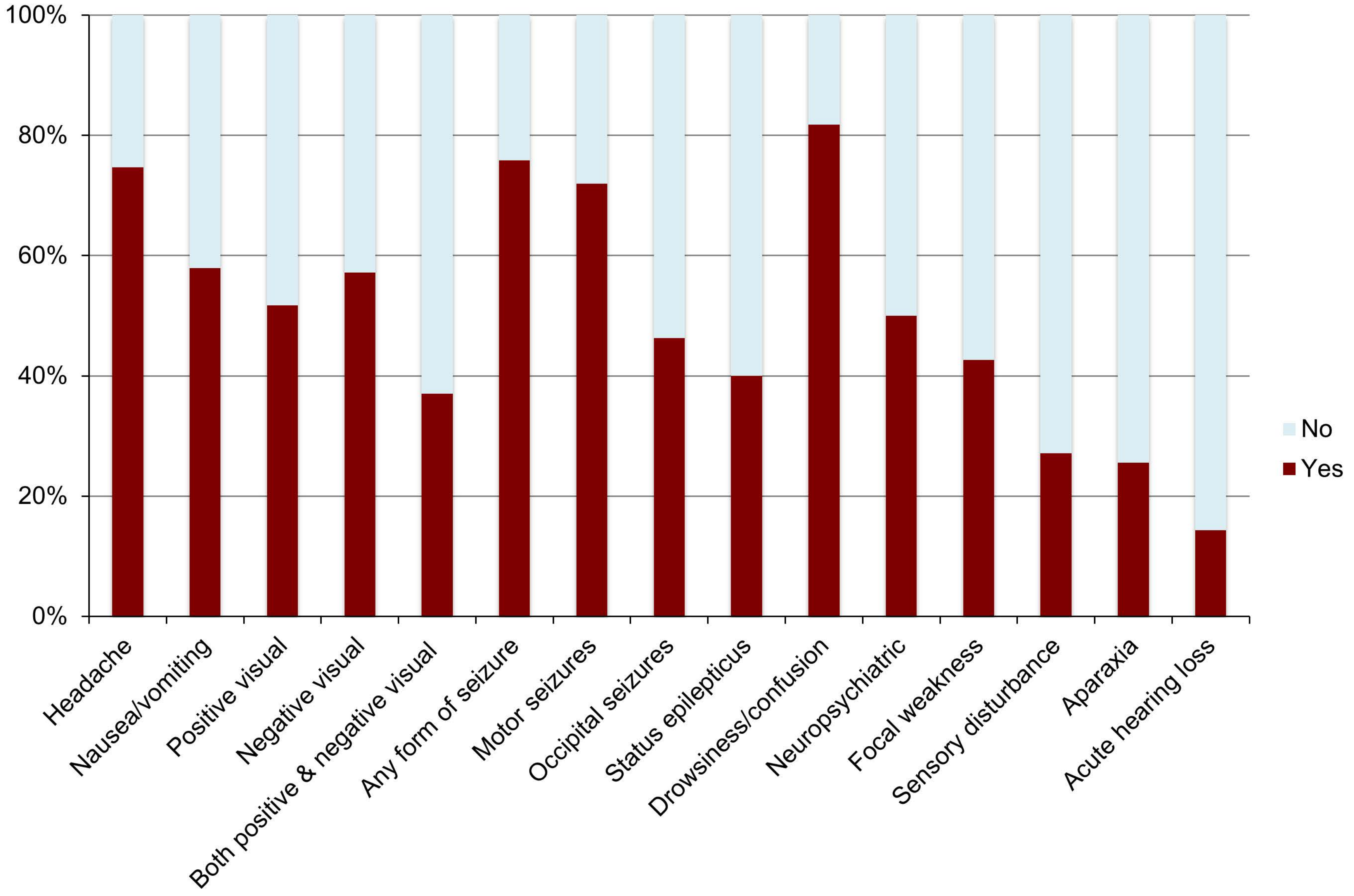

Supplemental Figure 3

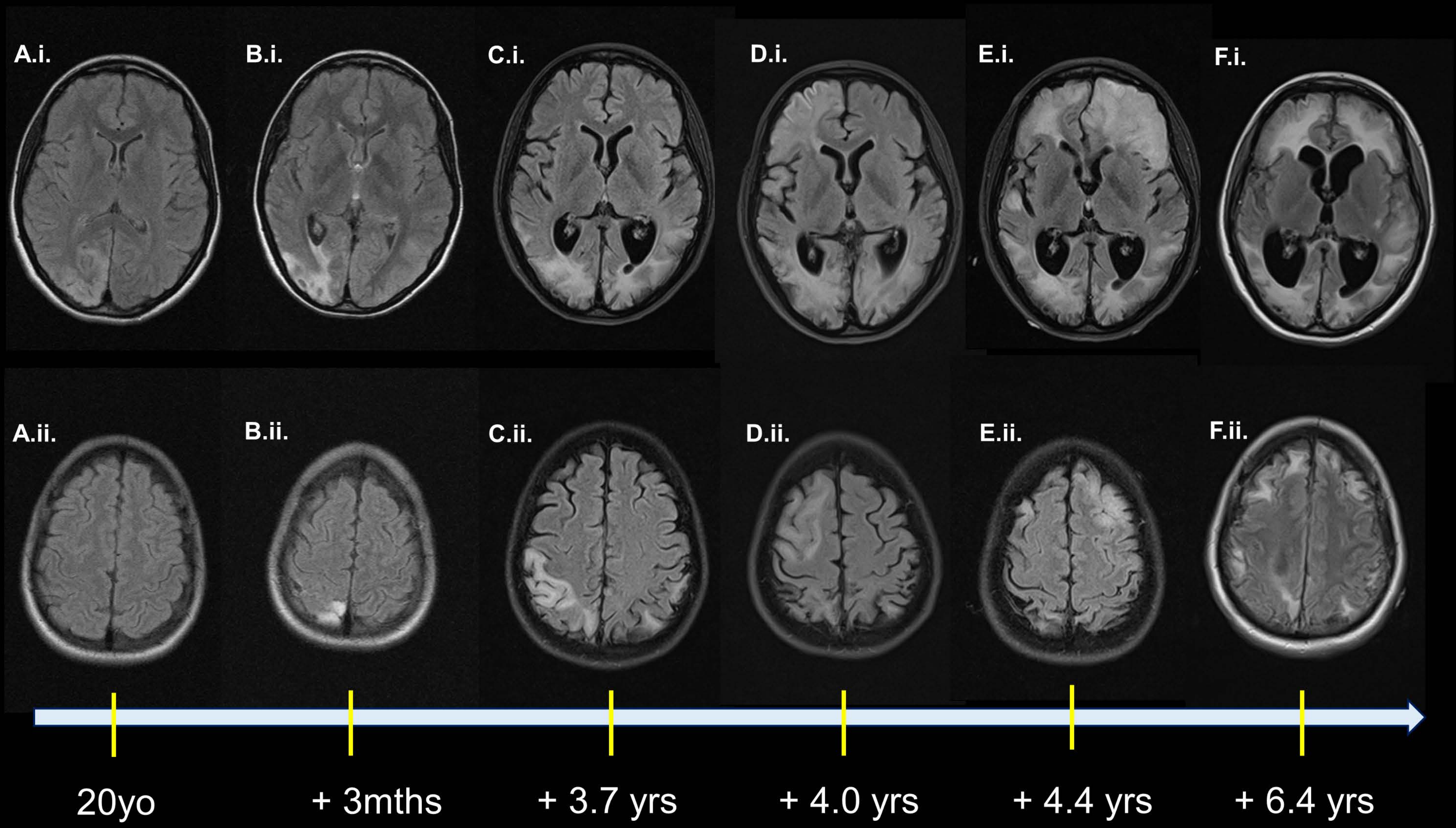

Supplemental Figure 4

(A)

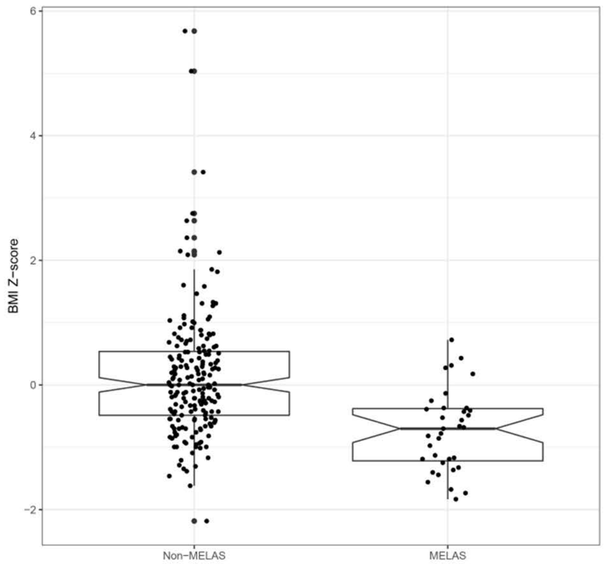

Median	0.002	-0.700
IQR	-0.486; 0.538	-1.218; -0.379
Mean	0.132	-0.735
95% CI	-0.01; 0.27	-0.96; -0.51

(B)

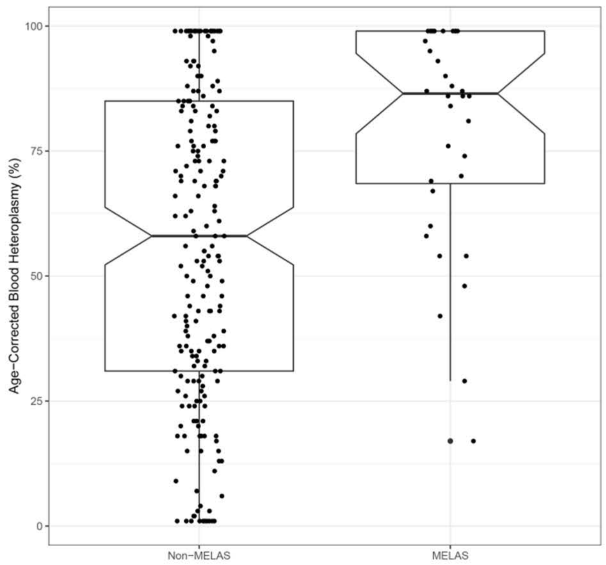

Median	58%	87%
IQR	31%; 85%	69%; 99%
Mean	57%	80%
95% CI	53%; 61%	72%; 87%

(C)

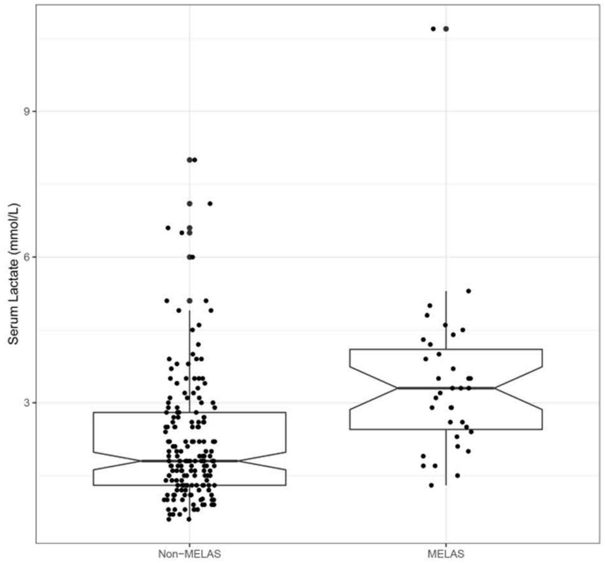

Median	1.8	3.3
IQR	1.3; 2.8	2.5; 4.1
Mean	2.2	3.4
95% CI	2.0; 2.4	2.9; 4.0

(D)

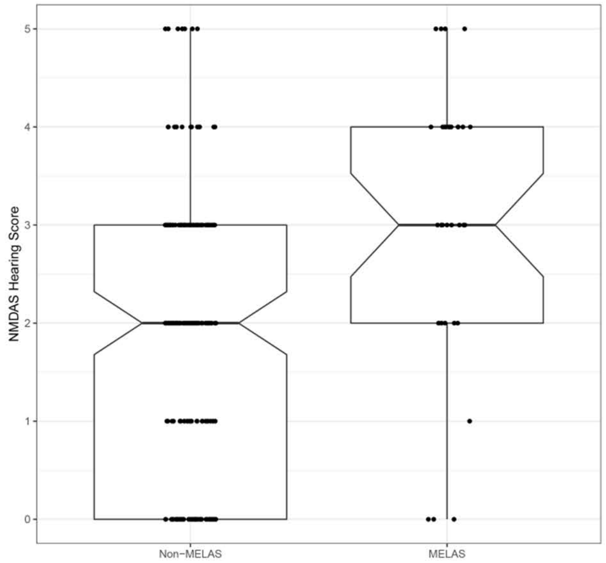

Median	2	3
IQR	0; 3	2; 4
Mean	1.8	3.1
95% CI	1.6; 2.0	2.6; 3.5

Supplemental Figure 5

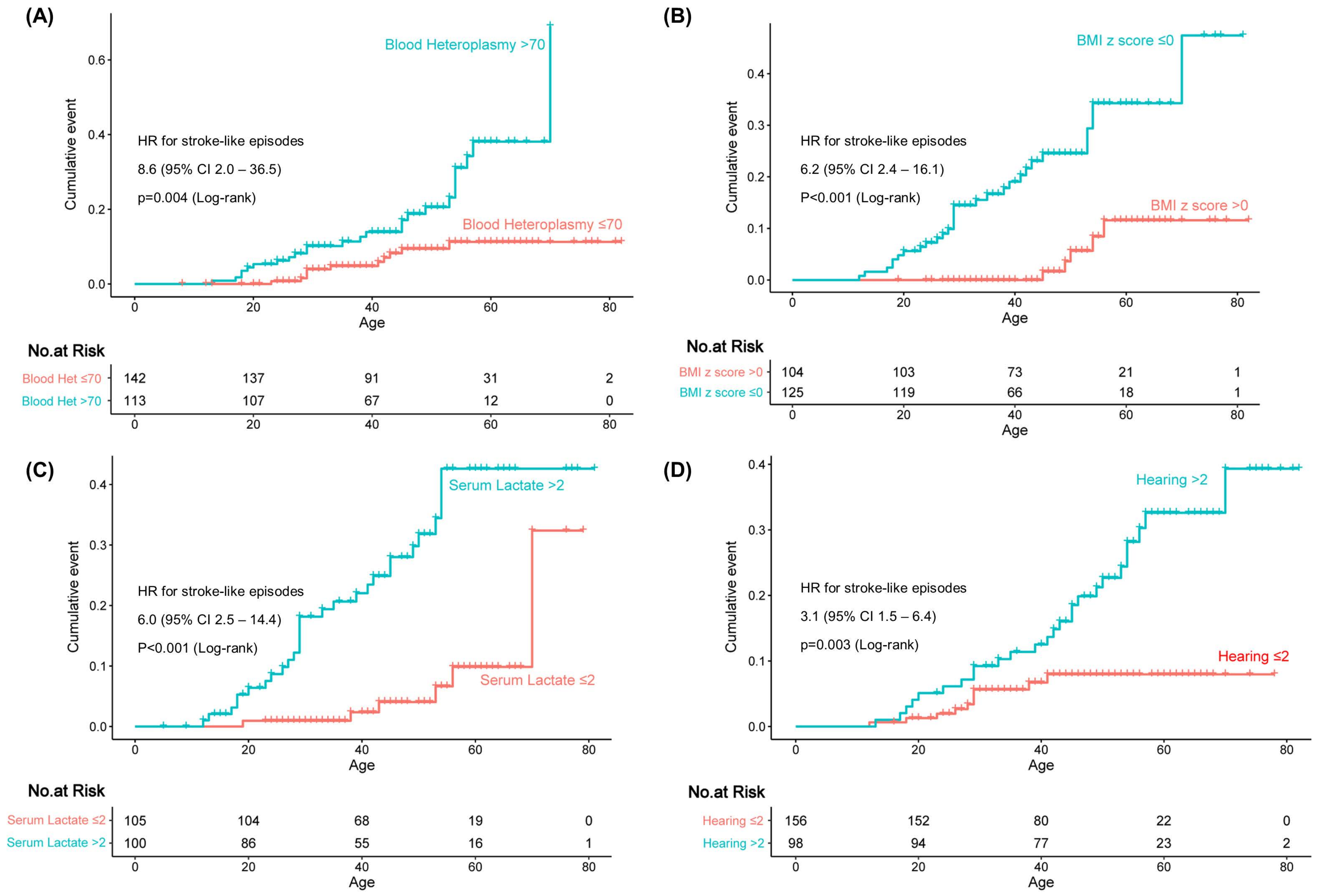

Supplemental Figure 6

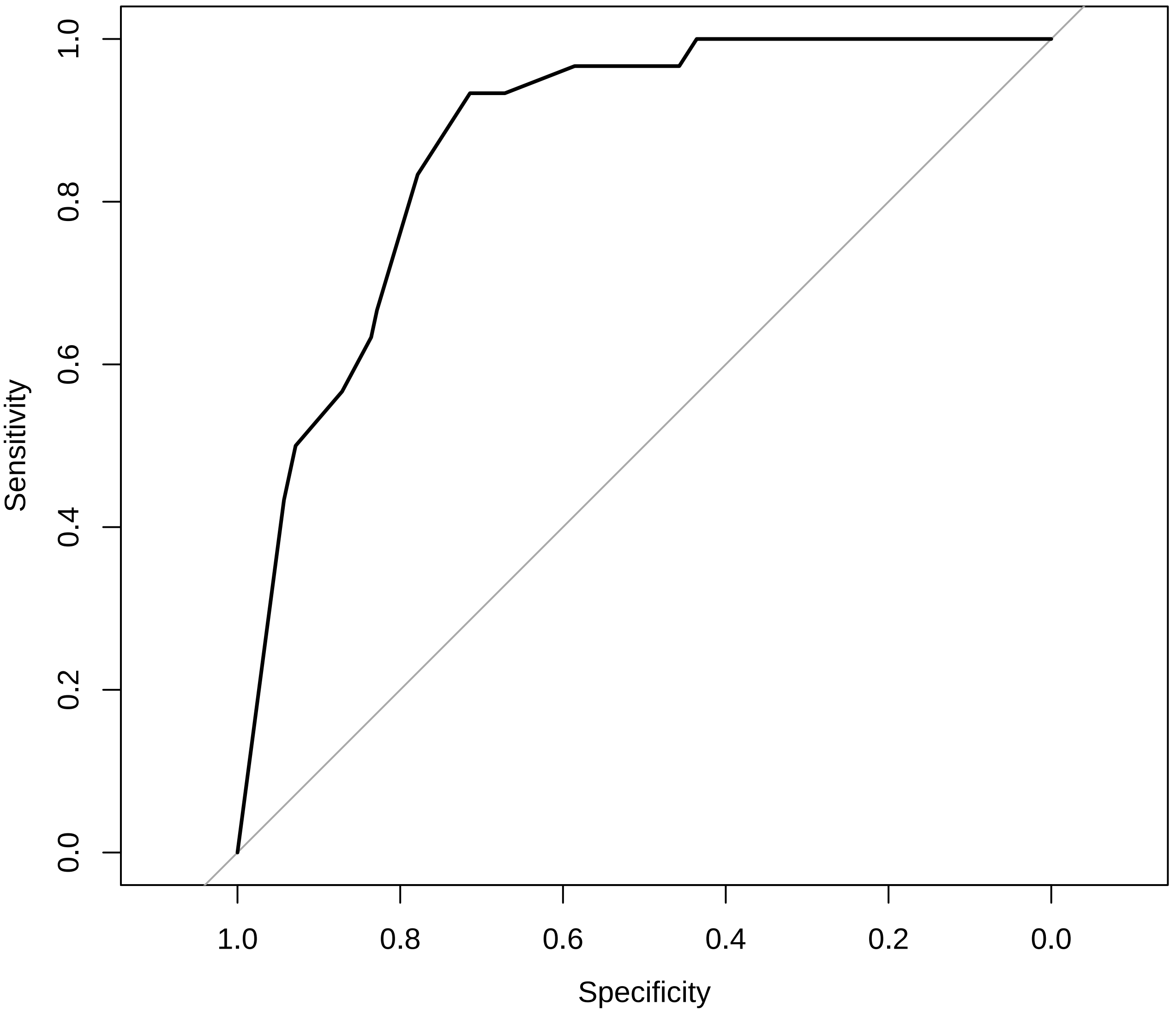

Supplemental Figure 7

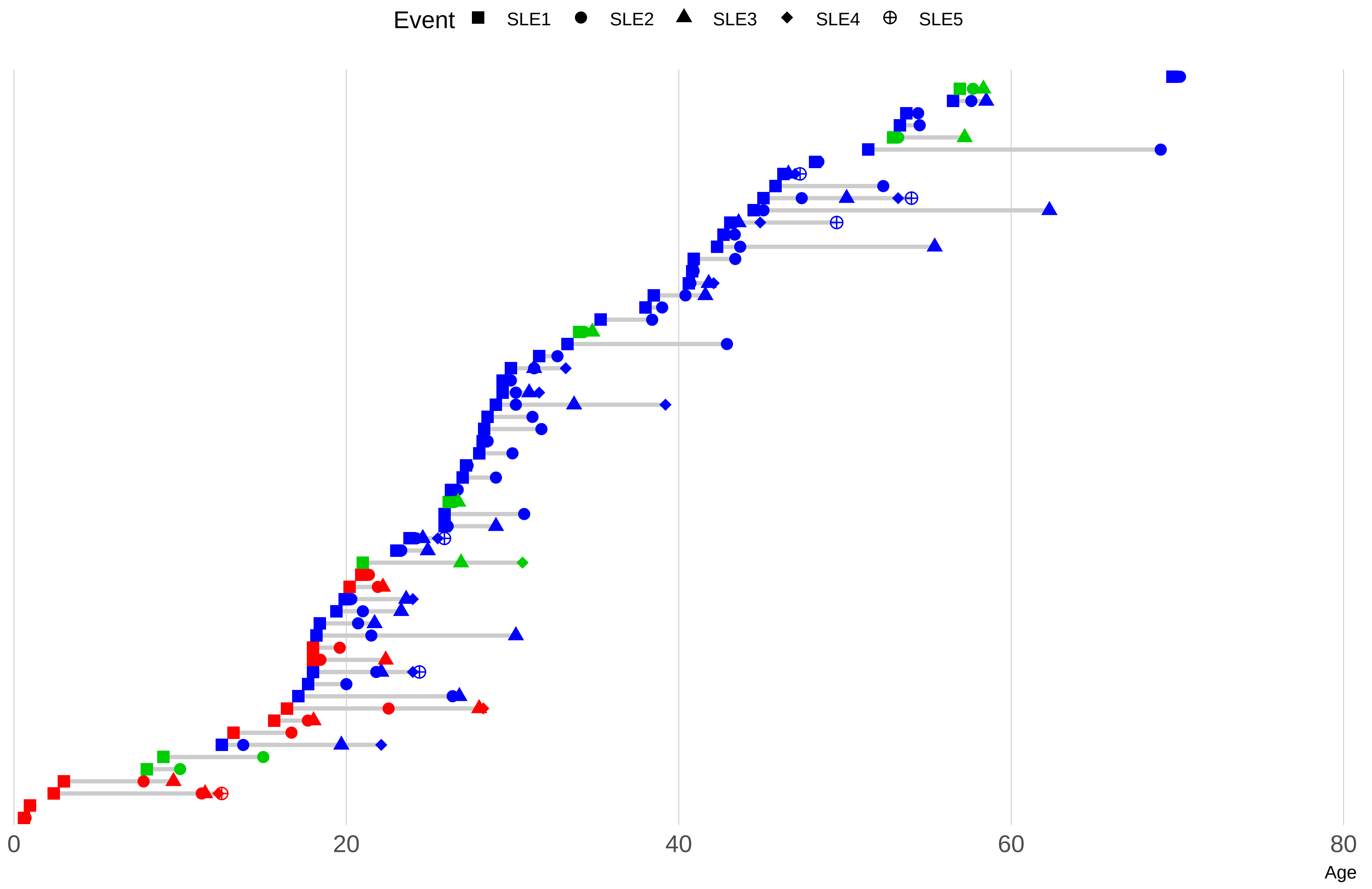

Supplemental Figure 8

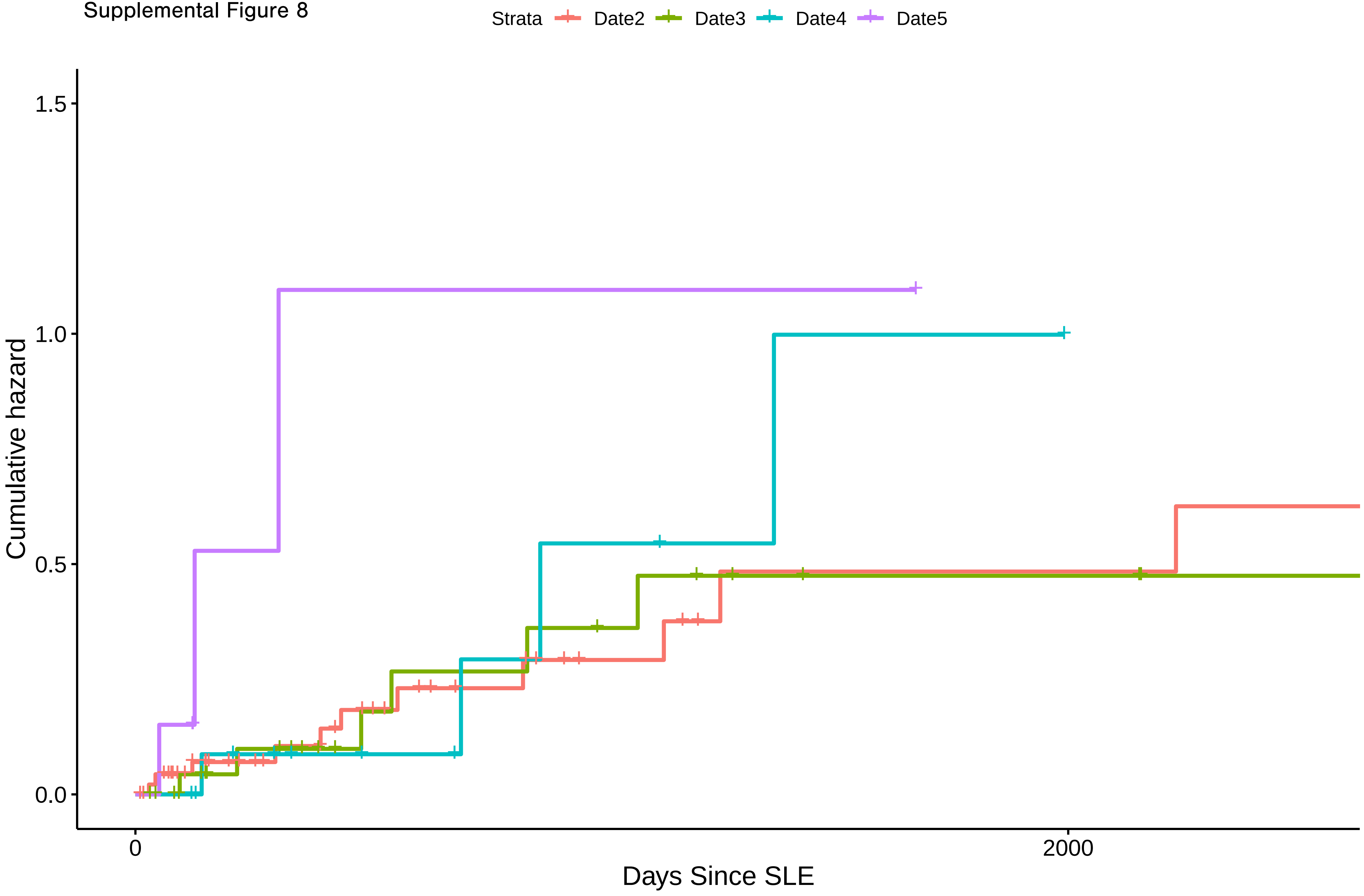

Supplemental Figure 9

A.i.

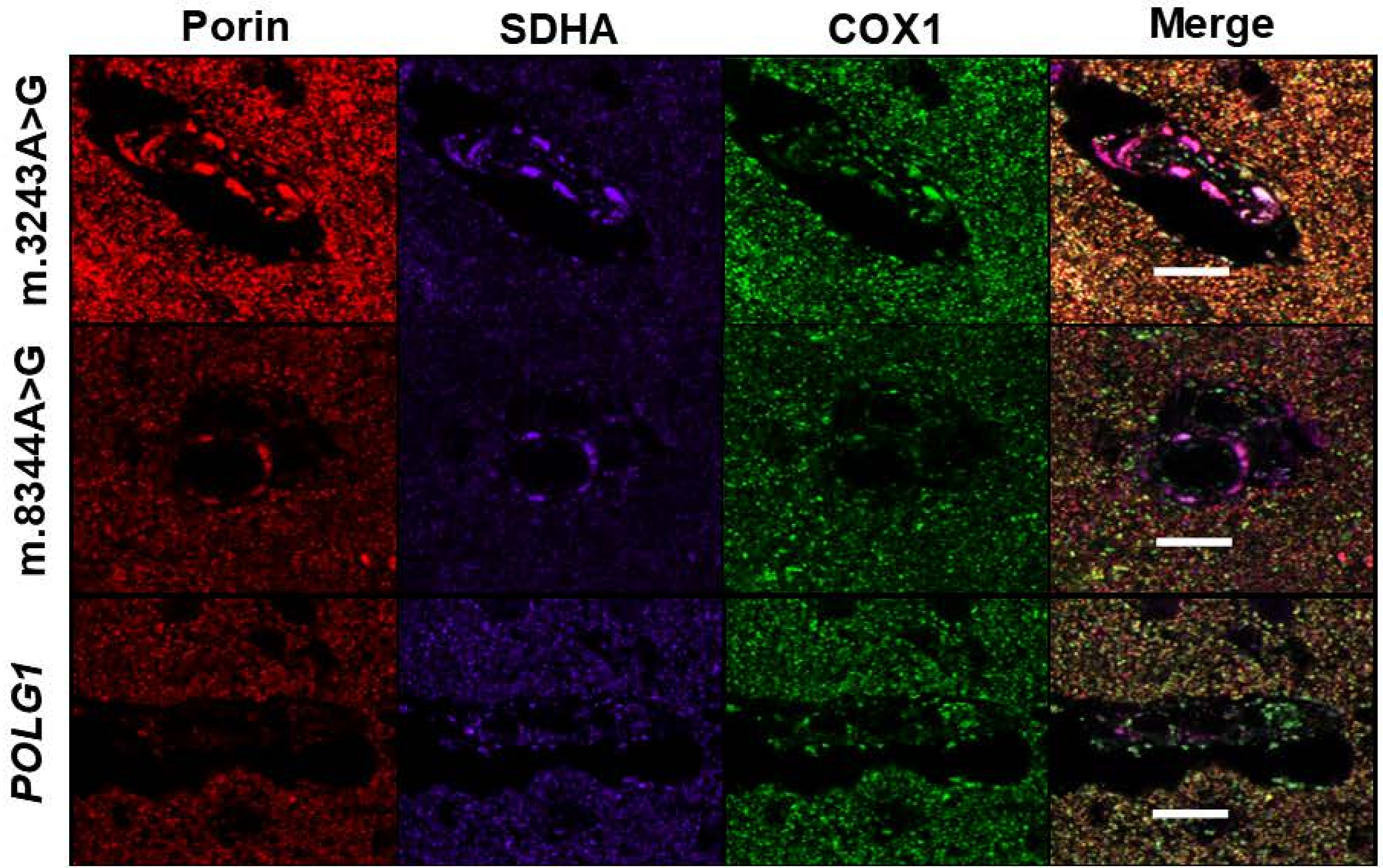

A.ii.

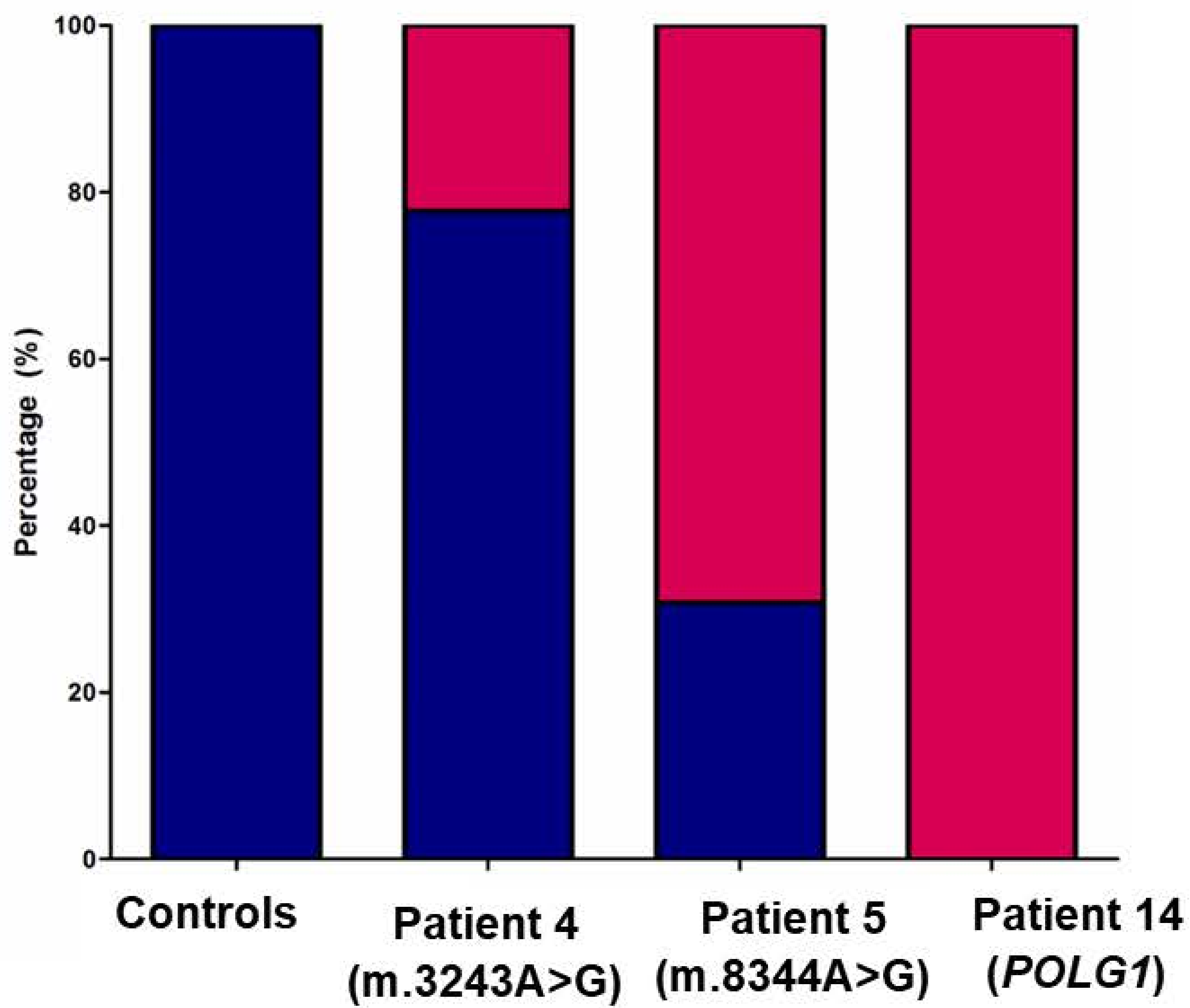

Supplement: awab353_Supplementary_Data [file awab353_supplementary_data.zip › brain-2021-00937-File010.pdf]
